# The photochemical mechanism of a B_12_-dependent photoreceptor protein

**DOI:** 10.1038/ncomms8907

**Published:** 2015-08-12

**Authors:** Roger J. Kutta, Samantha J. O. Hardman, Linus O. Johannissen, Bruno Bellina, Hanan L. Messiha, Juan Manuel Ortiz-Guerrero, Montserrat Elías-Arnanz, S. Padmanabhan, Perdita Barran, Nigel S. Scrutton, Alex R. Jones

**Affiliations:** 1 School of Chemistry, The University of Manchester, Oxford Road, Manchester M13 9PL, UK; 2 Photon Science Institute, The University of Manchester, Alan Turing Building, Oxford Road, Manchester M13 9PL, UK; 3 SYNBIOCHEM, Manchester Institute of Biotechnology, The University of Manchester, 131 Princess Street, Manchester M1 7DN, UK; 4 Faculty of Life Sciences, The University of Manchester, Carys Bannister Building, Dover Street, Manchester M13 9PL, UK; 5 Department of Genetics and Microbiology, Area of Genetics (Unidad Asociada al Instituto de Química Física–Consejo Superior de Investigaciones Científicas), Faculty of Biology, Universidad de Murcia, 30100 Murcia, Spain; 6 Instituto de Química Física ‘Rocasolano', Consejo Superior de Investigaciones Científicas, 28006 Madrid, Spain

## Abstract

The coenzyme B_12_-dependent photoreceptor protein, CarH, is a bacterial transcriptional regulator that controls the biosynthesis of carotenoids in response to light. On binding of coenzyme B_12_ the monomeric apoprotein forms tetramers in the dark, which bind operator DNA thus blocking transcription. Under illumination the CarH tetramer dissociates, weakening its affinity for DNA and allowing transcription. The mechanism by which this occurs is unknown. Here we describe the photochemistry in CarH that ultimately triggers tetramer dissociation; it proceeds via a cob(III)alamin intermediate, which then forms a stable adduct with the protein. This pathway is without precedent and our data suggest it is independent of the radical chemistry common to both coenzyme B_12_ enzymology and its known photochemistry. It provides a mechanistic foundation for the emerging field of B_12_ photobiology and will serve to inform the development of a new class of optogenetic tool for the control of gene expression.

## Abstract (web summary)

Coenzyme B12 traditionally acts as cofactor to light-independent metabolic enzymes in bacteria and humans. Here, Kutta *et al.* present a time-resolved photochemical description of a B12-dependent photoreceptor protein, which represents a mechanistic foundation for B12 photobiology.

## Graphical abstract

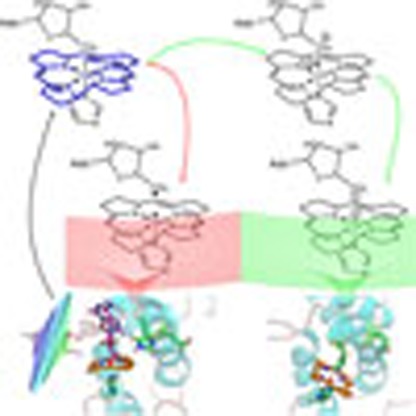

Coenzyme B_12_ (5′-deoxyadenosylcobalamin, AdoCbl) is a large, structurally complex organometallic molecule and a biologically active derivative of vitamin B_12_ (ref. 1). The mechanistic biochemistry of various AdoCbl-dependent enzymes is well described^2^, and recent crystal structures have provided insight into the binding of AdoCbl to riboswitches, which controls the expression of genes involved in its biosynthesis and transport^3^,^4^. However, little is known about the photochemical mechanism that facilitates the role of AdoCbl as a photoreceptor chromophore^5^. Although the photosensitivity of AdoCbl has been known for many years^6^, until recently this photochemistry was considered to serve no physiological function. Free in aqueous solution, photoexcitation of AdoCbl leads to Co−C bond homolysis generating the cob(II)alamin/5′-deoxyadenosyl radical pair^7^. This is the same radical pair that triggers catalysis on substrate binding to most AdoCbl-dependent enzymes. The photolysis of AdoCbl has therefore been used to investigate the influence of cofactor structure and environment on the enzymatic reaction dynamics^8^.

Many bacteria respond to light-induced oxidative stress by synthesizing carotenoids^9^. The prospect of B_12_-dependent photobiology arose when light-dependent activation of the genes that lead to carotenoid biosynthesis in the bacterium *Myxococcus xanthus* was shown to be B_12_-dependent^10^. The transcriptional repressor, CarH, requires B_12_ for activity *in vivo*^11^, and specifically AdoCbl for oligomerization, DNA binding and light-triggered tetramer dissociation *in vitro*^5^,^12^. Over 200 CarH-like proteins have been identified in various bacterial genomes (InterPro database, EBI)^5^. Furthermore, B_12_ has been proposed to act as chromophore in the protein AerR, which controls the light-induced synthesis of bacteriochlorophyll in *Rhodobacter capsulatus* and other photosynthetic bacteria^13^. It appears, therefore, that B_12_ defines a new class of photoreceptor protein.

Here we present the detailed photochemical mechanism over femtoseconds to seconds for CarH from *Thermus thermophilus*. These transient absorption data reveal a chemical pathway quite distinct from anything observed previously for a B_12_ molecule and one that appears to be independent of the radical intermediates so closely associated with AdoCbl biochemistry and photochemistry. This work therefore introduces a new branch of B_12_ biochemistry and provides a mechanistic foundation for the emerging field of AdoCbl-dependent photobiology.

## Results

### Photoexcitation of CarH results in a highly stable adduct

**Figure 1:**
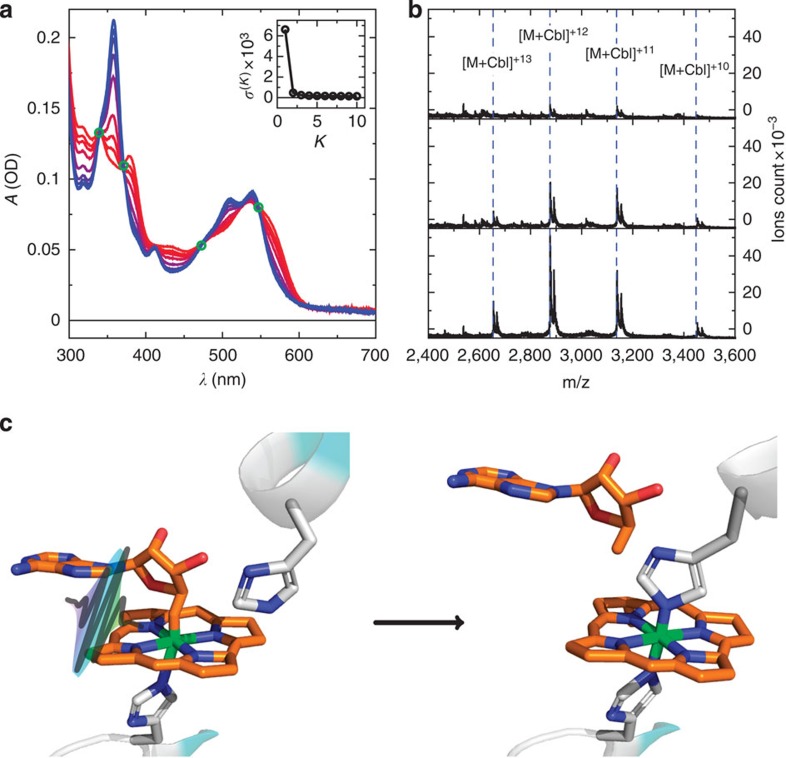
The photoconversion of CarH. (**a**) Ultraviolet–visible spectra showing the photoconversion of the CarH tetramer (CarH-GS, red spectra) to the monomeric ‘light-state' photoproduct (CarH-LS, blue spectra) under stepwise illumination (pulse duration=100 ms, *λ*_max_=530 nm). Isosbestic points (green circles) suggest a transition from a single state to another, supported by SVD-based rank analysis (inset), which shows that only two components are required to represent the data (Supplementary Fig. 1). (**b**) Nanoelectrospray ionization mass spectra of CarH-GS injected in the dark (top), after continuous illumination with white light for ∼1 min (middle), and illumination for 2 min (bottom). Signals of the general form [M+Cbl]^n+^ are annotated, where 10≤*n*≤13, M=33,142 Da and Cbl=1,329 Da. The total molecular weight is 34,471 Da, which corresponds to the predicted mass of CarH-LS. The secondary peak (higher mass) in each signal is consistent between both CarH-GS and -LS, and is likely a result of an adduct formed during protein purification. (**c**) Proposed photochemical conversion of base-off AdoCbl in CarH-GS (with incident photon) to a cobalamin adduct with an active-site residue (likely either H132 or H142), which represents the CarH-LS.

The ultraviolet–visible absorption spectra acquired during stepwise illumination of the CarH tetramer ground state (CarH-GS) show a clean transition directly to a single cobalamin photoproduct (or light state, CarH-LS) without other significant contributions (Fig. 1a). This is supported by singular value decomposition (SVD)-based rank analysis of the data (Fig. 1a, inset), the decomposed mole fraction versus time profiles and pure species spectra from which are shown in Supplementary Fig. 1a,b, respectively. The CarH-LS spectrum has pronounced structure at 478 and 509 nm (ref. 5), and closely resembles the previously published spectrum of a cobalamin with imidazole as upper and lower axial ligands^14^. The AdoCbl-binding domains of CarH proteins share sequence similarity with methylcobalamin (MeCbl)-dependent methionine synthase and AdoCbl-dependent methylmalonyl CoA mutase, both of which bind the cofactor base-off with an active-site histidine coordinated to the lower axial position of the Co. Indeed, mutation of a conserved histidine (H177A) in CarH that aligns with those that form the lower axial linkage in these B_12_-dependent enzymes has been shown to stop AdoCbl binding and tetramer formation^5^. Moreover, the equivalent H193A mutation in CarH from *M. xanthus* abolishes function *in vivo*^11^. The putative B_12_-dependent photoreceptor protein AerR from *Rhodobacter capsulatus* is also thought to bind base-off with a lower axial histidine. Although the details of its photochemical mechanism are not yet known, after illumination, AerR has been shown to bind the cofactor via a strong covalent linkage between the Co and a second upper axial histidine, a bond that is sustained after treatment with 0.04 M HCl (ref. 13).

Our attempts to isolate the cobalamin bound to CarH-LS by heating to 90 °C and by treatment with 6 M urea were unsuccessful (confirmed by size-exclusion chromatography (SEC)), consistent with a similar covalent linkage to that in AerR being formed between cobalamin and the protein during the photochemical reaction in CarH. This is supported by further data from SEC that show that cobalamin bound to CarH-LS cannot be displaced with free AdoCbl in fivefold excess, whereas hydroxocobalamin bound to CarH can be, with subsequent formation of the CarH-GS tetramer (Supplementary Fig. 2). Both AdoCbl and OHCbl bind to CarH with reasonably high affinities (*K*_D_∼250 and ∼5 μM, respectively; Supplementary Fig. 3). The lower affinity of CarH for OHCbl could be owing to the lack of favourable binding in this case between the protein and the 5′-deoxyadenosyl. OHCbl has been previously shown by SEC to bind to CarH, but unlike AdoCbl binding this does not result in tetramer formation^12^ and there is no evidence that it forms the CarH-LS under illumination (Supplementary Fig. 4). The latter point is perhaps unsurprising when one considers the fact that photoexcitation of free OHCbl does not result in dissociation of the upper axial OH (ref. 15). The aqueous buffer we used in our illumination experiments with OHCbl-bound CarH was at pH 7.5, where OHCbl is mostly in the form H_2_OCbl. H_2_OCbl has also been found to not undergo appreciable photolysis^16^.

We confirmed the formation of a highly stable adduct between protein and cofactor by native mass spectrometry. On continuous illumination of CarH-GS with white light, strong signals grow in that indicate an accumulation of CarH-LS, the mass of which correspond to an adduct between the CarH monomer and AdoCbl minus its upper axial 5′-deoxyadenosyl ligand (Fig. 1b). CarH-LS was then mass selected using the quadrupole of a Synapt G2 mass spectrometer and fragmented using argon as collision gas in the trap cell region (Supplementary Fig. 5). When no collision energy is applied we only observe the mass distribution of the precursor ion. When the collision energy is increased we observe the formation of two main additional ions corresponding to the loss of cobalamin from the precursor ion. These data support the assignment of a protein/cobalamin adduct in CarH-LS. Owing to its spectral similarity with that of cobalamin with imidazole as upper and lower axial ligands^14^, we speculate that this adduct is formed between the cobalamin and a second active-site histidine (Fig. 1c).

**Figure 2:**
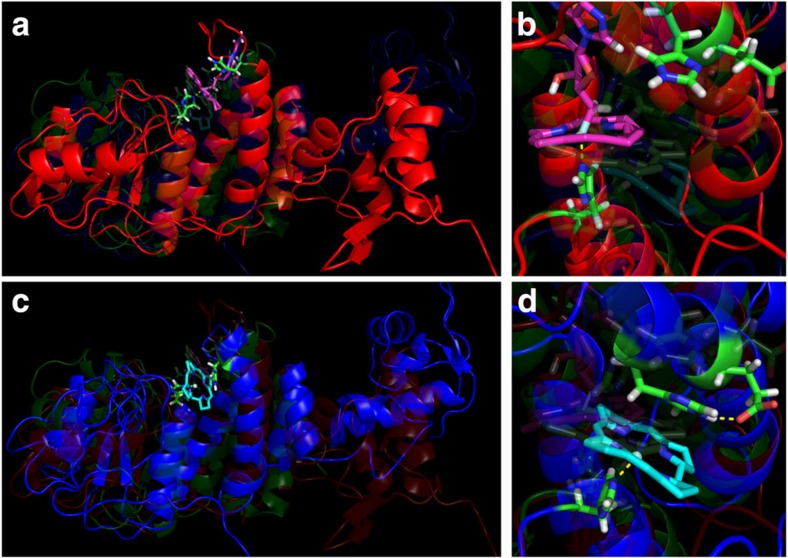
Molecular dynamics simulations of the CarH photoconversion. Each structure shown is based on a homology model (Supplementary Figs 6–8); AdoCbl is represented in magenta and active site residues H177 (lower axial coordination), H132 and E129 in green. Molecular dynamics simulations (Supplementary Figs 9,10) were conducted with (**a**,**b**, red structure) and without (**c**,**d**, blue structure) the 5′-desoxyadenosyl. The snapshots of CarH are representative structures at the minimum of root mean square deviation plots for 10 ns molecular dynamics simulations following 4 ns of relaxation after alignment to the heavy atoms of the cobalamin-binding domain (Supplementary Fig. 9a,c (black lines)).

To explore the possible nature of the adduct, we generated a homology model of the CarH-GS structure (Supplementary Figs 6–8). The AdoCbl-binding site (Supplementary Fig. 8) shows that the chromophore is bound ‘base-off', with the 5,6-dimethylbenzimidazole displaced by H177. Two histidine residues (H132 and H142) are also adjacent to the upper face of AdoCbl, either of which might plausibly form a covalent linkage with the Co. Molecular dynamics simulations were run on this structural model in the presence and absence of the upper axial 5′-deoxyadenosyl ligand (Supplementary Figs 9,10). Removal of this ligand produces a significant structural change in the protein, which results in a switch in conformation of the corrin ring from the starting structure (Fig. 2a,b) to a position where H132 is directly above the Co (Fig. 2c,d). A glutamate residue (E129) is well placed to abstract a proton from H132 if necessary for adduct formation (Fig. 2d and Supplementary Fig. 8). The photoconversion is therefore likely to be between base-off AdoCbl, which is bound via a lower axial histidine to CarH-GS, and an adduct similar to that observed in AerR, where the 5′-deoxyadenosyl has been displaced by either H142 or H132 (that is, CarH-LS, Fig. 1c). Although our homology model and molecular dynamics simulations (Supplementary Figs 6–10) favour adduct formation with H132, the precise identity of CarH-LS needs to be established, for example, by crystallography. Adduct formation is followed by dissociation of the CarH tetramer, thus weakening its affinity for DNA and allowing transcription. To identify the various transient intermediates between the CarH-GS and CarH-LS states we have used time-resolved spectroscopic methods measuring ultraviolet–visible absorbance changes between femtoseconds and seconds.

### CarH significantly alters the photochemistry of AdoCbl

The ultrafast photochemical dynamics of AdoCbl, in solution and bound to B_12_-dependent enzymes, are well described^17^,^18^,^19^. After photoexcitation of AdoCbl in aqueous solution, two excited state intermediates have been fully resolved that decay to a base-off cobalamin intermediate within tens of ps. This dissociates into a base-on cob(II)alamin/5′-deoxyadenosyl radical pair in ∼100 ps, the majority of which (∼80%) recombine to the ground state within 10 ns. The photochemistry of AdoCbl bound to B_12_-dependent enzymes such as glutamate mutase^20^,^21^ or ethanolamine ammonia lyase^22^ involves the same radical pair. However, the state that immediately precedes Co−C bond homolysis has been assigned to a cob(III)alamin-like metal-to-ligand charge transfer (MLCT) state, much like that observed during photolysis of MeCbl (ref. 23). Moreover, the quantum yield of longer-lived radicals decreases (to 5–10%) when AdoCbl is confined to an enzyme active site^20^,^24^.

**Figure 3:**
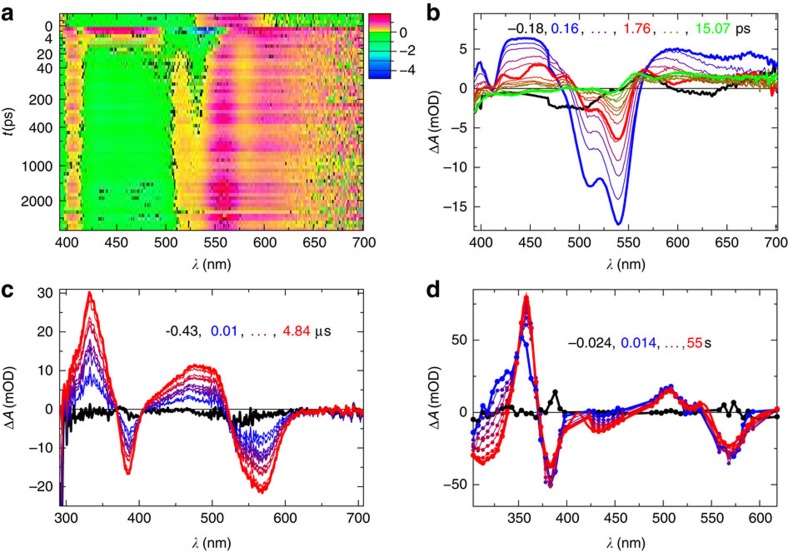
The transient photoresponse of CarH. (**a**) False colour plot of the raw data (that is, uncorrected for background data) transient absorption difference spectra that represent the photodynamics of CarH <3 ns (yellow to red, positive amplitudes; green to blue, negative amplitudes). (**b**) Examples of raw data transient absorption difference spectra recorded using an ultrafast pump–probe spectrometer up to 15 ps following the photoexcitation of CarH by a fs-pulsed laser. (**c**) Examples of raw data transient absorption difference spectra recorded using a streak camera up to 5 μs following the photoexcitation of CarH by a ns-pulsed laser. (**d**) Examples of raw data transient absorption difference spectra recorded using an ultraviolet–visible spectrometer up to 55 s following the photoexcitation of CarH by a ms-pulsed LED. (**b**–**d**) Black lines represent background data, blue lines the early time data and red lines later time data; the green line in (**b**) represents the contribution of the component that lives from 15 ps to beyond the data acquisition window (that is, ≫3 ns).

The photodynamics of AdoCbl over fs–s are strikingly different when bound to CarH (Fig. 3a–d). False colour representations of the ultraviolet–visible transient absorption difference spectra acquired at time delays up to 3 ns after photoexcitation of CarH-GS are shown in Fig. 3a. Individual examples of raw data difference spectra from the first 15 ps (Fig. 3b), which are not corrected for the pre-*t*_0_ signal, show that signal appears within about 160 fs, comprising two broad, positive absorption bands at wavelengths <490 and >560 nm, and a ground state bleach (that is, a negative signal that represents the loss of a ground-state absorption band) with peaks at 510 and 540 nm. These features decay into a highly structured difference spectrum within 15 ps (Fig. 3b, green spectrum). Apart from the recovery of the bleach at 540 nm over ∼400 ps, this spectrum remains constant from 15 ps over the entire acquisition window (Fig. 3a). The linear dependence of this signal (corrected for the pre-*t*_0_ signal) on laser power confirms that it is the result of single-photon excitation (Supplementary Fig. 11a–c). A significant contribution from multiphoton processes would cause the laser power dependence to deviate from linearity and can therefore be ruled out.

**Figure 4:**
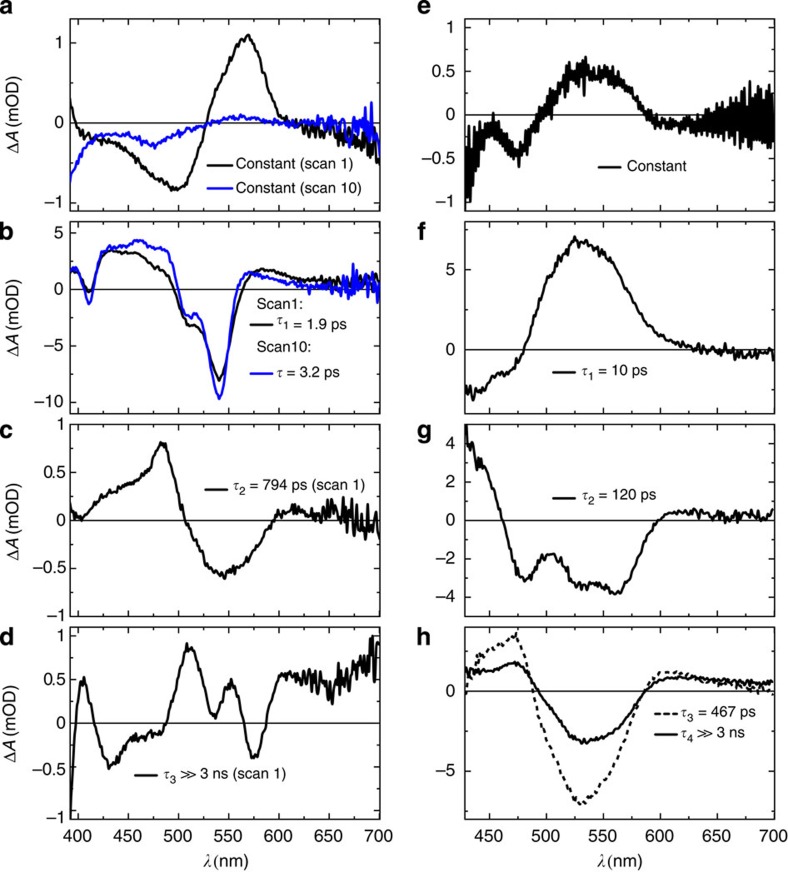
Decay-associated difference spectra of the ultrafast photoresponse. Comparison of DADS that result from SVD and global analysis of ultrafast transient absorption data acquired between −1.5 ps and ∼3 ns following photoexcitation of CarH-GS (**a**–**d**) and free AdoCbl (**e**–**h**). The constant functions (**a**,**e**) describe the baseline. The remaining black lines show the dynamic components from fresh sample with lifetimes *τ*_1–3_ (**b**–**d**: 1.9 ps, 794 ps and ≫3 ns, respectively), for CarH and *τ*_1–4_ (**f**–**h**: 10 ps, 120 ps, 467 ps (dashed) and ≫3 ns, respectively) for AdoCbl. Blue lines are DADS from sample that has mostly converted to the monomeric light state (CarH-LS); they show one constant (**a**) and one dynamic component with lifetime *τ*=3.2 ps (**b**).

The effect of CarH on the ultrafast photoresponse of AdoCbl is well illustrated by comparing the decay-associated difference spectra (DADS) from the CarH data to those from an equivalent data set from free AdoCbl (black DADS in Fig. 4a–d,e–h, respectively). DADS were identified by globally fitting the raw data (for CarH, the data in Fig. 3a) to a sum of exponentials. The number of exponentials used was determined by using SVD-based rank analysis^25^. A good global fit to the data from CarH-GS required one constant component (Fig. 4a) and three dynamic components with lifetimes, *τ*_1–3_ (Fig. 4b–d). The resulting DADS represent the amplitudes of the exponential components and are used to generate species-associated spectra (SAS) for the various reaction intermediates using a model. The lifetime associated with each DADS is used to calculate the rate constant of each chemical conversion (for SAS and rates see the next section). The DADS with the shortest lifetime from the CarH data set (Fig. 4b *τ*_1_=1.9 ps) is quite distinct from that of free AdoCbl (Fig. 4f) and more closely resembles the transient difference spectra observed following photoexcitation of non-alkyl cobalamins such as CNCbl (ref. 16). The homolysis product, cob(II)alamin, is formed (Fig. 4c) but not via a base-off intermediate as observed for free AdoCbl (Fig. 4g). Moreover, these radicals are much shorter lived (*τ*_2_=794 ps) than those observed for free AdoCbl photolysis (Fig. 4h), with no evidence of any remaining beyond ∼1 ns. In fact, the DADS with a lifetime beyond the data acquisition window (Fig. 4d, *τ*_3_≫3 ns) closely resembles the difference spectrum that corresponds to the MLCT state known to precede homolysis during the photolysis of MeCbl (ref. 23) and AdoCbl bound to glutamate mutase^21^. By contrast, there is no evidence within the 3ns time window of Co−C bond homolysis following the formation of this MLCT state in AdoCbl bound to CarH.

If a series of repeated, −1.5 ps to 3 ns data acquisition scans are recorded on the same sample, the observed signal changes significantly as the sequence progresses (Supplementary Fig. 12a–c). Example spectral changes from the first and the tenth scans of a sequence are shown in Supplementary Fig. 12d–g, respectively. In the latter case, the photodynamics are complete within 15 ps with no evidence of the longer-lived (*τ*_3_≫3 ns), structured spectrum typical of the MLCT state observed in the earlier scans. This is consistent with the irreversible formation of photoproduct, with the photoresponse of accumulated CarH-LS dominating later scans. The DADS from a later scan (blue DADS, Fig. 4b, and black DADS Supplementary Fig. 13) also bears a close resemblance to the excited state difference spectrum of CNCbl (ref. 16), and similarly decays completely to the ground state within ps.

There is significant signal in the pre-excitation baseline from CarH-GS (that is, in the data acquired before the pump pulse from the laser, Fig. 3a). It is not visible in data from CarH-LS (Supplementary Fig. 12c) and corresponds to the constant component from the analysis of the early scans (black DADS, Fig. 4a). The repetition rate of the laser is 1 kHz; this signal therefore represents an intermediate with a lifetime longer than 1 ms. Our simulations of a hypothetical pump–probe transient absorption experiment (Supplementary Fig. 14) show that species with lifetimes longer than the time difference between two sequential probe pulses are expected to result in an inverse transient absorption signal before the subsequent laser excitation (Supplementary Fig. 15). On a similar (ms) timescale, a population of radical pairs produced by the photolysis of free and enzyme-bound AdoCbl are known to undergo spin-selective and magnetically sensitive recombination^17^,^20^,^24^,^26^,^27^,^28^. To further investigate the origin of this pre-excitation signal, transient absorption difference spectra were acquired ns–μs after the photoexcitation of CarH-GS. They show positive and negative features that grow throughout the acquisition time, with peak shifts at wavelengths <400 nm (Fig. 3c). SVD and global analysis give two similar but statistically distinct dynamic components (Supplementary Fig. 16), suggesting the same chemical species in a changing environment. The first component has a lifetime of *τ*_1_=0.6 μs and, as predicted by our simulations, the second is an inversion of the constant component from the ultrafast data (black trace, Fig. 4a). This component therefore corresponds to the pre-excitation baseline signal from the ultrafast data (Figs 3a, 4a), and has a lifetime of >1 ms. Unlike intermediates from free AdoCbl photolysis on a similar timescale, however, it does not resemble difference spectrum that represents the conversion from cob(III)alamin to the cob(II)alamin radical (that is, Co−C bond homolysis). Spectral changes continue on ms–s timescale (Fig. 3d), with two further DADS, which are similar to the CarH-LS ground state between 500 and 600 nm (Supplementary Fig. 17a). However, the absorption at 333 nm does increase initially, followed by a bi-exponential decay, and at 358 nm, increases and continues to grow bi-exponentially into the final, CarH-LS spectrum at 55 s (Supplementary Fig. 17b), after which no further changes are observed. Much like the ns–μs intermediates, the spectral changes over ms–s are consistent with a single chemical species in a changing environment.

### CarH does not employ a radical mechanism

**Figure 5:**
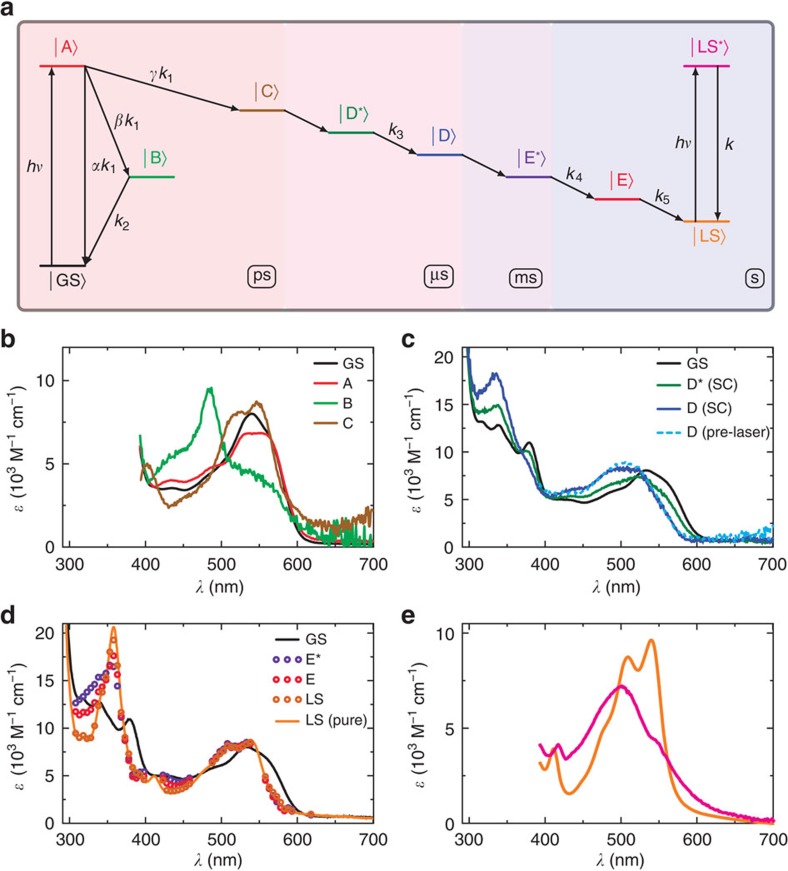
Model and species-associated spectra representing the CarH reaction intermediates. (**a**) Schematic representation of the model used to generate the SAS from the ground-state (GS) spectrum and DADS (Supplementary Figs 18–21), which correspond to the excited states, reaction intermediates (**a**–**e**) and photoproduct (or ‘light state', LS). Each intermediate decays with rate constant, *k*_1–5_ and *α*, *β* and *γ* represent branching ratios. ps, μs, ms and s time domains are indicated. (**b**–**e**) Coloured SAS correspond to the colour code of each intermediate in (**a**), and the black line in each is the GS spectrum. (**b**) ps intermediates observed by ultrafast transient absorption. (**c**) ns–μs intermediates observed in the streak camera data; the pre-excitation baseline signal from the ultrafast pump–probe experiments (pale blue, dashed line) closely resembles intermediate, D (dark blue line). (**d**) ms–s intermediates; the final state, LS (brown dots), corresponds to the spectrum known^5^ for the CarH-LS (pure, orange line). (**e**) SAS of the CarH (photoproduct) excited state (magenta line) built from the ground-state spectrum (orange line) and corresponding DADS.

**Figure 6:**
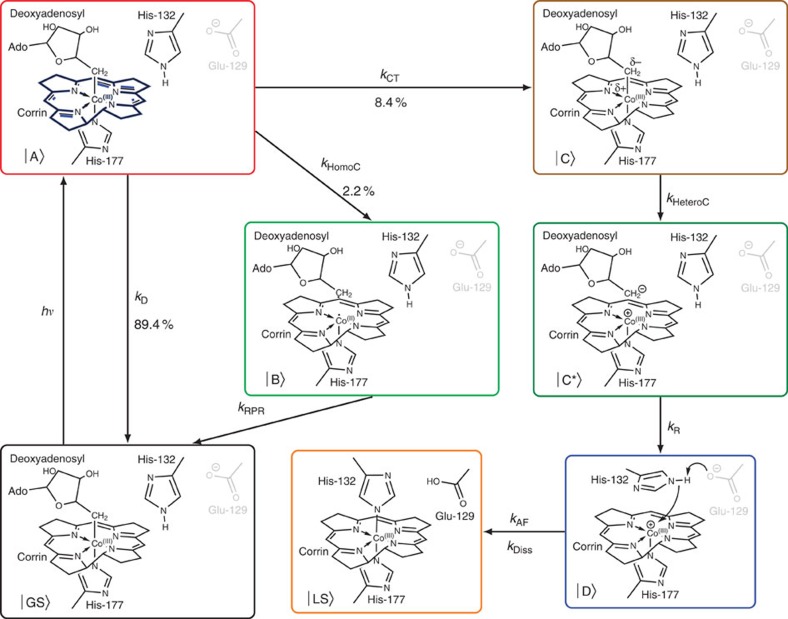
Proposed photochemical mechanism of CarH. The coloured boxes and letters correspond to the colour-coded ground state (GS), intermediates (**A**–**D**) and product (or ‘light state', LS) in Fig. 5. Intermediates E* and E in Fig. 5 have not been shown here because they represent the adduct between protein and the cobalamin before complete dissociation of the tetramer and therefore resemble LS. AdoCbl is represented with a simplified corrin ring for clarity, with the Co coordinated by a lower axial H177 from the protein. The involvement of residues H132 and E129 are those favoured by our molecular dynamic simulations based on our homology model of the CarH structure (Fig. 2). After photoexcitation (*hν*) of the GS, each step proceeds with a rate constant, *k*_*i*_, where *i*: CT, charge transfer; HomoC, homolytic cleavage; HeteroC, heterolytic cleavage; R, rearrangement; AF, adduct formation; Diss, tetramer dissociation. See main text for discussion of the mechanism.

The model in Fig. 5a was applied to the DADS resulting from the global analysis over fs–s. From this modelling we generated a SAS for each intermediate observed (Fig. 3b–e) from which we propose the photochemical mechanism in Fig. 6. Photoexcitation of CarH-GS gives an initial excited state A, the signal from which decays with the rate *k*_1_. From our proposed model, A either relaxes to the ground state with rate *αk*_1_=*k*_D_=0.471 × 10^12^ s^−1^ or decays into one of two intermediates B or C with rates *βk*_1_=*k*_HomoC_=0.012 × 10^12^ s^−1^ and *γk*_1_=*k*_CT_=0.044 × 10^12^ s^−1^, respectively (Fig. 5a). *α*, *β* and *γ* are branching ratios, where *α*+*β*+*γ*=1. The green SAS of intermediate B in Fig. 5b is distinctive of the Co−C bond homolysis product, the cob(II)alamin radical (Fig. 6, green box)^18^. This is a non-productive channel, with a recombination rate *k*_2_=*k*_RPR_=1.26 × 10^9^ s^−1^, after which all of the radical pairs recombine to the ground state. The brown SAS of C in Fig. 5b closely resembles the estimated spectrum of the MLCT state reported previously^21^,^23^, where the Co(III) effectively receives dative coordination from an anionic ligand (Fig. 6, brown box). This intermediate has a lifetime of ≫3 ns and leads to a productive channel ending in the photoproduct. The branching ratios in our model are *α*=0.894, *β*=0.022 and *γ*=0.084. They suggest that, following a single-fs laser pulse, the majority of excited states decay to the ground state, with only 8.4% leading to the photoproduct and tetramer dissociation. A relatively low yield of productive channel would be consistent with the need for carotenoid biosynthesis only under high light intensity conditions, when the bacteria that express CarH are likely to experience significant photo-oxidative stress^5^,^11^.

The dark green SAS of D* in Fig. 5c represents the next observed intermediate formed from C between 3 and 15 ns with an approximate rate *k*_HeteroC_∼0.1 × 10^9^ s^−1^. It resembles neither the spectrum of cob(II)alamin nor cob(I)alamin, the Co−C bond dissociation products observed in reactions catalysed by AdoCbl and MeCbl-dependent enzymes, respectively. The spectrum has lost some of the structure evident at ∼550 nm in the preceding MLCT state (C in Fig. 5b), but still retains cob(III)alamin character. D* decays into the dark blue SAS of intermediate D (Fig. 5c) with a rate *k*_3_=*k*_R_=1.79 × 10^6^ s^−1^. This decay involves a blue shift in the broad feature around 500–550 nm and a slight growth of the peak at ∼350 nm. Such relatively subtle spectral shifts are consistent with a change in the chromophore environment, with a blue shift suggesting it has become more hydrophobic (as observed for the chromophore in active-site variants of AdoCbl-dependent ethanolamine ammonia lyase)^29^. Consistent with the analysis of the ns–μs data is Co−C bond heterolysis to give five-coordinate cob(III)alamin and the 5′-deoxyadenosyl anion (Fig. 6, D*, dark green box), the latter of which is then displaced by the residues involved in adduct formation (Fig. 6, D, dark blue box). Generation of the MLCT state of the preceding intermediate (C) has shifted electron density onto the ligand, which might favour generation of the 5′-deoxyadenosyl anion. Displacement of this polar moiety after dissociation would also be expected to result in the spectral blue shift observed on transition from D* to D. The molecular dynamic simulations (Fig. 2 and Supplementary Figs 9,10) also support this; they reveal a switch in conformation of the corrin ring to a position that displaces the recently dissociated 5′-deoxyadenosyl and favours adduct formation. The 5′-deoxyadenosyl anion can then undergo β-hydride elimination to generate 4′,5′-anhydroadenosine, which has been recently identified as the only counterpart photoproduct to the CarH-LS (ref. 30). Although electron transfer from the 5′-deoxyadenosyl anion to cob(III)alamin to generate the radical pair might be considered possible, there are no spectral intermediates consistent with this happening. A potential explanation is that it is precluded by the kinetics of the structural rearrangement that follows 5′-deoxyadenosyl dissociation.

An alternative assignment of spectral intermediates D* and D is hydridocobalamin^31^, a poorly characterized six-coordinate, cob(III)alamin species with an upper axial H. We were prompted to consider this possibility by the identification of 4′,5′-anhydroadenosine as the sole photoproduct following aerobic photolysis of CarH (ref. 30). This product has been previously observed after the thermal homolysis of AdoCbl, where β-hydrogen transfer from the 5′-deoxyadenosyl radical to the cob(II)alamin radical represents a possible reactive fate to give 4′,5′-anhydroadenosine and hydridocobalamin^32^,^33^. We have no evidence of radicals subsequent to the MLCT intermediate C in the CarH photoresponse. However, it cannot yet be ruled out that Co−C cleavage and H transfer in CarH somehow occurs in a concerted fashion or that a hydride is transferred from the 5′-deoxyadenosyl anion to the 5-coordinate cob(III)alamin. Either way, the 4′,5′-anhydroadenosine could then be displaced by the structural changes described above, followed by substitution of the upper axial H to form the CarH-LS adduct. Because of the resemblance of D from the ns–μs data to the SAS from the pre*-*excitation baseline signal from the ultrafast data (Fig. 5c, pale blue dashed) it is likely that this intermediate lives for >1 ms.

The SAS with lifetimes of ms–s (Fig. 5d, E*, purple dots and E, dark red dots) are almost identical to that of the CarH-LS between 500 and 600 nm, suggesting that after intermediate D, cobalamin does indeed form the stable adduct with an active-site residue (Fig. 6, orange box, *k*_AF_∼0.1 × 10^3^ s^−1^). Owing to the close resemblance of the CarH-LS spectrum (Fig. 3a) to that of a cobalamin with imidazole as upper and lower axial ligands^14^, we speculate that this is a histidine, and our homology model of the CarH structure (Supplementary Figs 6–8) places both H132 or H142 close by. Molecular dynamics simulations (Supplementary Figs 9,10) favour H132 and show that an active-site glutamate E129 is well positioned in case proton abstraction from H132 is necessary for nucleophilic attack (Figs 2b,d, 6, dark blue box). As stated above, the identity of the adduct needs to be confirmed experimentally. Despite the lack of significant changes between 500 and 600 nm, there are spectral changes in the blue and ultraviolet between the SAS representing intermediates E*→E→LS (Fig. 5d). These are likely to be a result of changes to the cofactor environment caused by structural changes in the protein that are triggered by adduct formation and which ultimately result in dissociation of the CarH tetramer into monomers. On the basis of the slow rates of interconversion between each of these states (*k*_4_=*k*_Diss_=1.71 s^−1^ and *k*_5_=*k*_Diss'_=0.06 s^−1^) from the global fit of the ms–s data, we propose two alternative pathways: (i) structural changes internal to each monomer (*k*_Diss_) that trigger complete tetramer dissociation (*k*_Diss'_); or (ii) dissociation into dimers (*k*_Diss_) followed by dissociation into monomers (*k*_Diss′_). Further study is required to distinguish between these dissociative pathways. Finally, the SAS of the excited state of CarH-LS (Fig. 5e) bears a close resemblance to the estimated excited state spectrum of CNCbl, which has been previously attributed to a ligand to metal charge transfer state^16^. For a justification of the model chosen in Fig. 5a from the various different models explored, see Supplementary Figs 18–21 and the Supplementary Methods section.

## Discussion

We have described the detailed photochemical mechanism over the fs–s that follow photoexcitation of a new class of photoreceptor protein that is dependent on AdoCbl. In doing so we have revealed that AdoCbl is capable of diverse biochemistry to enable its increasingly diverse known biology. In summary, after photoexcitation of AdoCbl, electron density is moved away from the Co towards the upper axial 5′-deoxyadenosyl ligand forming a MLCT state, which then leads to the dissociation of the Co−C bond. The dissociation products are 4′,5′-anhydroadenosine^30^ and a cob(III)alamin species that could potentially be the five-coordinate product of heterolysis or six-coordinate hydridocobalamin^31^. Nucleophilic attack by an active-site residue, likely either H132 or H142, then forms a stable adduct with the cob(III)alamin, the irreversible formation of which is followed by dissociation of the CarH tetramer. Therefore, our data suggest that the productive pathway of CarH does not proceed via the cob(II)alamin/5′-deoxyadenosyl radical pair intermediates so closely associated with both AdoCbl enzymology and photochemistry^8^. The fact that formation of the stable cobalamin adduct appears to be irreversible is perhaps surprising considering the high energetic cost of AdoCbl biosynthesis^34^. However, although CarH proteins only bind AdoCbl to form a tetramer *in vitro*^5^, exogenously supplying other cobalamins such as CNCbl, MeCbl or OHCbl *in vivo* is sufficient for CarH repressor activity^11^. This implies the existence of intracellular machinery that can replace the upper axial 5′-deoxyadenosyl after photoconversion and thus recycle the cofactor, possibly after proteolysis and release of cobalamin. The adenosyltransferase enzyme capable of activating cobalamins such as CNCbl (ref. 34) is a likely candidate^5^.

We have thus shown that CarH tunes AdoCbl photochemistry to facilitate the response of bacteria to photo-oxidative stress. It also appears that other B_12_-dependent bacterial proteins (for example, AerR) play an intimate role in the light-dependent control of photosynthetic genes^13^. A compelling new area of molecular and mechanistic photobiology is therefore beginning to emerge based on B_12_ as photoactive chromophore. Our findings represent the first photochemical mechanism for a B_12_-dependent photoreceptor protein and should serve to guide the development of this class of photoreceptors as optogenetic tools for the control of gene expression in cells and organisms.

*Note added in proof:* crystal structures of the CarH-GS tetramer and CarH-LS monomer states are about to be published^40^. These structures reveal the bis-His linkage involving H132 in CarH-LS and the large conformational change associated with its formation from the CarH-GS tetramer.

## Methods

### Materials

Coenzyme B_12_ (>98% Sigma) was used as purchased without further purification. The plasmid, pET15b-CarH, encoding CarH from *Thermus thermophilus* was transformed into *Escherichia coli* and the apoprotein monomer overexpressed. The His_6_-tagged protein was purified under native conditions using Talon metal affinity resin (GE Healthcare) and imidazole elution^5^,^12^. All spectroscopic experiments were conducted in 50 mM phosphate buffer, 150 mM NaCl, pH 7.5.

### Preparation of cobalamin-bound CarH samples

All steps were conducted under dim red light. CarH tends to precipitate if its concentration is brought above ∼30 μM before AdoCbl binding and tetramer formation. A dilute solution of the apoprotein monomer (<1 μM) was therefore incubated with a large excess of AdoCbl or OHCbl (∼250 μM) for 10–15 min. Unbound cobalalmin was then removed from the resulting CarH tetramer dark state using a CentriPure P100 gel filtration column (emp BIOTECH). Each sample of the cobalamin-bound CarH was then concentrated as required using centrifugal filtration devices (10 kDa molecular weight cutoff).

### Stationary ultraviolet–visible absorption spectroscopy

Stationary ultraviolet–visible absorption spectra were recorded using an Agilent Cary 50 UV-Vis Spectrophotometer. Samples of the CarH-GS tetramer or OHCbl-bound CarH were illuminated using a pulsed, high-power light-emitting diode (LED; M530L3, Thorlabs) with *λ*_max_=530 nm. The 100ms rectangular excitation pulse (2 mJ) was collimated using an anti-reflection-coated aspheric lens (Thorlabs), and delivered along the 2mm pathlength of the cuvette orthogonal to the detection beam. After each excitation an absorption spectrum was recorded resulting in a sequence of spectra over illumination time. The sample volume was 150 μl with an optical density over 1 cm of ∼0.1 at the excitation wavelength. The entire sample volume was therefore homogeneously illuminated, avoiding possible effects of diffusion during the recording of the spectra.

### Analysis of the photoconversion of CarH-GS to CarH-LS

The data matrix **A** shown in Fig. 1a represents the overall photoconversion of CarH-GS to CarH-LS, and was decomposed into its time profiles and corresponding spectra using a method employed previously^35^. First, the number of principle components in **A** was determined using SVD-based rank analysis. Second, a model matrix **X** was generated based on known features such as isosbestic points, known spectra and assumptions about the characteristics of the unknown spectra (for example, wavelengths where the extinction coefficient is assumed to be zero). Finally, the model matrix **X** allows the reconstruction of the species spectra **S** and their corresponding concentration time profiles **C** via:


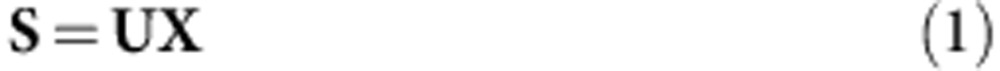



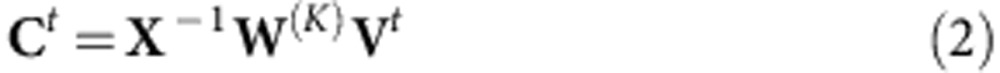


where the matrices **U**, **V** and **W**^(*K*)^ are the result of the SVD, with *K* principle components used to reconstruct the data matrix **A**:


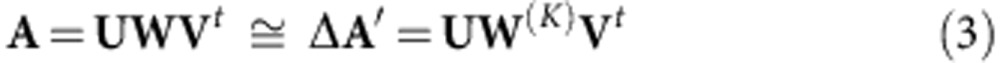


SVD analysis of the CarH photoconversion shows that only two principle components are necessary to represent the data (inset in Fig. 1a). The species spectra and time profiles were therefore calculated using these components and the following assumptions: (i) the CarH-GS species spectrum is the first spectrum of the sequence; (ii) the final spectrum of the sequence is the CarH-LS species spectrum contaminated by the CarH-GS spectrum. Accounting for ∼2% of the ground state in the final spectrum allowed the sum of both concentration time profiles to be 1 over the entire temporal range. The mole fraction versus time profiles and pure species spectra are shown in Supplementary Fig. 1a,b, respectively.

### Size-exclusion chromatography

To assess the stability of the protein/cobalamin adduct in CarH-LS, we attempted to displace the cobalamin with an excess of free AdoCbl. The following samples were separately incubated before eluting each by SEC: hydroxocobalamin (OHCbl)-bound CarH with fivefold excess of AdoCbl (black). CarH-LS with fivefold excess of AdoCbl (red).

All procedures were conducted under safe light. The elution profiles recorded at 280 and 522 nm for each pre-incubation condition are shown in Supplementary Fig. 2a,b, respectively.

### Cobalamin-binding titrations

The *K*_D_ of AdoCbl and OHCbl binding to CarH were estimated using a spectral method. Samples were prepared containing different concentrations of CarH apoprotein (up to 27 μM) and a constant concentration (5 μM) of each cobalamin. An ultraviolet–visible absorption spectrum was acquired for each concentration of apoprotein used (Supplementary Fig. 3a,c) and the spectral evolution was analysed using a similar method to that described above for the photoconversion of the CarH-GS. Briefly, the data matrix was decomposed into its principle species spectra and corresponding mole fraction profiles (Supplementary Fig. 3b,d). Here the mole fraction profiles show a typical binding curve of the following chemical equilibrium:


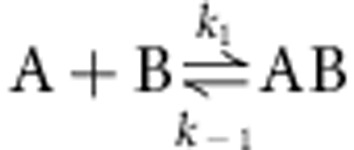


where,


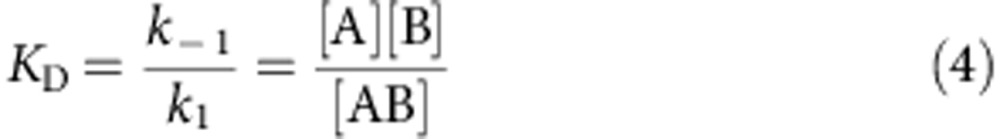


The amount of complex AB, *x*_AB_, is dependent on [A] and [B], the concentration of the two binding partners, and is given by:


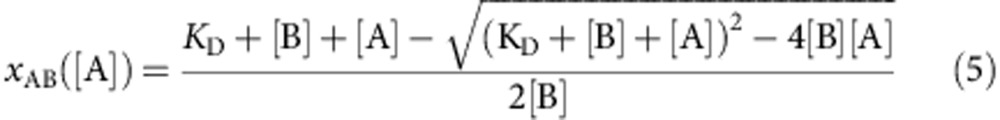


In each case [B] was assigned to that of the cobalamin and therefore held at 5 μM and the *K*_D_ for each cobalamin was determined by least squared fitting using the Levenberg–Marquardt algorithm.

### Mass spectrometry

Mass spectra were acquired using a Waters Synapt G2 mass spectrometer operating in time-of-flight mode. The tuning parameters were set to produce a mass resolving power of 20,000. The ions were produced using the Z-spray source and nanoelectrospray ionization. The spray voltage ranged from 1.3 to 1.8 kV and the source temperature was set to 80 °C. Collision-induced dissociation experiments were performed in the trap cell region of the Synapt using argon as a collision gas.

### CarH homology model

The programme MODELLER 9.11 was used to generate a homology model structure of full-length CarH (ref. 36). It is based on the available structures of methionine synthase from *E. coli* (PDB code: 1BMT)^37^, corrinoid (factor IIIm)-binding protein from *Moorella thermoacetica* (PDB code: 1Y80) and a transcriptional regulator of the MerR family from *Bacillus cereus* (PDB code: 3HH0). The structures 1BMT and 1Y80 include the CarH C-terminal B_12_-binding site, and 1BMT includes structural information about the linkage between the CarH photoreceptor and DNA-binding domains. Structural information about the CarH N-terminal DNA-binding domain is given by 3HH0. All three structures together cover the entire sequence of full-length CarH. An initial sequence alignment (Supplementary Fig. 6a,b) was made using the programme ClustalX 1.83 (Gap Opening: 10; Gap Extension: 0.2; Delay Divergent Sequence: 30%; Use Negative Matrix: yes; Protein Weight Matrix: BLO-SUM series)^38^,^39^. It shows CarH sequence identity of 16.5% to 1BMT, 22.8% to 1Y80 and 22.9% to 3HH0 (Supplementary Fig. 6a). The overall similarity in terms of the physicochemical properties of the amino acids is high for the corresponding domains (Supplementary Fig. 6b). Therefore, these templates are an acceptable basis for the model.

### Molecular dynamics simulations

Molecular dynamics simulations were performed on the CarH homology model structure using the Gromacs Package with the gromos53a6 force field, a solvation box of minimum 1 nm around the protein and periodic boundary conditions. After energy minimization the system was initially thermalized to 300 K for 100 ps using *NVT* dynamics, and the pressure was then equilibrated for 100 ps using *NPT* dynamics. The protein, cobalamin and 5′-deoxyadenosyl were constrained during these steps. All constraints and pressure couplings were then switched off and the system relaxed using *NPT* at 250, 280, 290 and 300 K for 1 ns each. Finally, 10 ns of *NPT* dynamics were run at 300 K. Representative structures were chosen as those with the smallest root mean squared deviations for the cobalamin and H132 heavy atoms relative to the average, following alignment to the heavy atoms of the cobalamin-binding domain (Supplementary Fig. 9).

### Ultrafast pump–probe transient absorption

All measurements were performed at room temperature using a Helios (Ultrafast Systems LLC) broadband pump–probe transient absorption spectrometer with an instrument response function of ∼170 fs. The Ti:sapphire amplifier (a hybrid Coherent Legend Elite-F-HE) is pumped by a Q-switched Nd:YLF laser (Positive Light Evolution-30) and seeded by a Ti:sapphire oscillator (Spectra-Physics Mai Tai). The amplifier output (1 kHz, 800 nm, with a typical pulse duration of ∼120 fs) is split to provide pump and probe beams. The pump beam is tuned using a non-collinear optical parametric amplifier (Light Conversion TOPAS-White) with a typical pulse duration of <70 fs. The concentration of CarH samples was adjusted to an optical density between 0.2 and 0.8 at the excitation wavelength (370 nm; 0.5 μJ per pulse; 2 mm pathlength). Data were recorded in a stirred quartz cuvette at time delays between −1.5 ps and 3 ns after excitation, the order of which was randomized. At each delay time, 1,000 single shots of pump and probe cycles were averaged.

### Simulations of pre-*t*_0_ signals from pump–probe experiments

To investigate how a long-lived signal might manifest in pump–probe transient absorption data acquired before the subsequent laser pulse, we performed a simulation of a hypothetical transient absorption experiment. In the simulation the frequency for collecting a single pair of signal and reference data sets was set to 0.5 kHz, and the simulation was based on four subsequent pump–probe cycles (8 ms). For simplicity, it is assumed that: (i) the sample volume does not change between each cycle; (ii) the hypothetical sample features only a ground 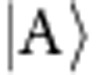 and excited state 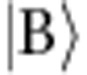; (iii) the sample consists of wavelengths at which the time evolution of the transmission arises only due to either pure absorbance of a new species (Supplementary Fig. 14a,b) or pure bleach of the ground-state absorbance (Supplementary Fig. 14c,d). The extinction coefficients of the ground and excited state were both set to 1 M^−1^ (cm)^−1^, and the optical pathlength was set to 1 cm. Photoexcitation of the sample was simulated by Gaussian-shaped laser pulses. The following differential equations were used to calculate the signals:


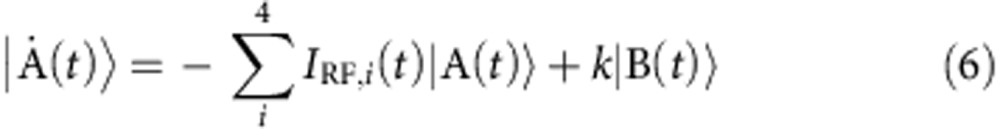



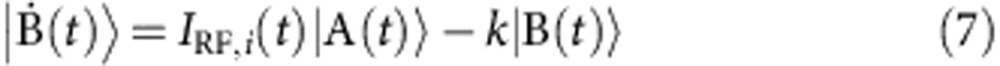


where 
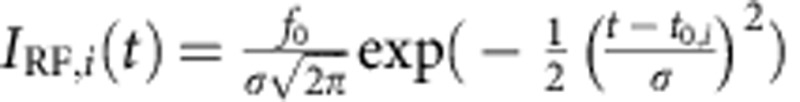
; *f*_0_=fraction of the ground state; *σ*=pulse width; and *t*_0,*i*_=time zero of pulse *i*.

Three simulations for three different rate constants, *k*_1_=10 (ms)^−1^, *k*_2_=3 (ms)^−1^ and *k*_3_=1 (ms)^−1^, of the excited state decay are shown in Supplementary Fig. 14.

Assuming that the total intensity applied to the sample is 1 at both wavelengths for pure absorption and pure bleach, the transmission values from the simulations are the measured intensities at these wavelengths. The transient absorption signals for a single pump–probe cycle can therefore be calculated using:


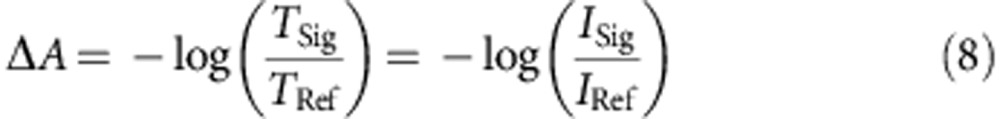


The results are shown in Supplementary Fig. 15. They demonstrate that species with lifetimes longer than the time difference between two probe pulses result in an inverse transient absorption signal before the subsequent laser excitation. They also show that such long-lived signals overlay with the transient absorption signals that evolve after the subsequent excitation pulse. The time window covered by the pump–probe experiment is typically two orders of magnitude smaller than the lifetime of the long-lived species. These contributions therefore manifest as a constant component in each data acquisition window, and these simulations therefore nicely illustrate the need to use a constant function over all acquisition wavelengths in the global fit analysis of our data.

### Nanosecond to microsecond transient absorption

The development and performance of the transient absorption spectrometer using streak camera detection employed here has been described in detail previously^25^. Briefly, the sample is flowed through a fused silica cuvette and the peristaltic pump was synchronized with the timing of the measuring process so that the sample was exchanged stepwise between each individual laser excitation. The sample is excited through a cylindrical lens (*f*=150 mm) along a 2-mm optical path by a single outcoupled laser flash (530 nm, 8 ns, 10 mJ) from an optical parametric oscillator pumped by a Nd:YAG laser (Surelite II, Continuum) at a repetition rate of 10 Hz. White light from a 150-W pulsed Xe flash lamp of ∼1ms duration (MSP-05, Müller Elektronik-Optik) is passed through the sample along a 10mm optical path and subsequently analysed by an imaging spectrograph (Bruker 200is, grating 100 grooves per mm) coupled to a streak camera (C7700, Hamamatsu Photonics). The data were registered by a charge-coupled device camera (ORCA-CR, Hamamatsu Photonics) and stored to a computer. Each transient absorption data set was calculated from four images taken with a frequency of 0.5 Hz in the following sequence: *D*_FL_—both flash lamp and laser; *D*_0_—without any incoming light; *D*_F_—only with the flash lamp; *D*_0_ repeated. This sequence is repeated 100 times, averaged and the transient absorption calculated as:


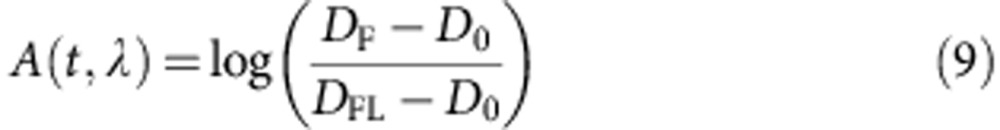


### Millisecond to second transient absorption

Samples were excited using the collimated, 10ms rectangular pulse (2 mJ) from a high-power LED (M530L3, Thorlabs) with *λ*_max_=530 nm. Sample exposure was conducted as described above for the stationary spectra to avoid effects of diffusion. Absorbance changes were recorded at single wavelengths ranging from 300 to 700 nm using an Agilent Cary 50 UV-Vis Spectrophotometer in 5 nm steps with a time resolution of 12.5 ms and time window of 55 s. The LED flash was manually triggered for each time trace and all data sets corrected to the same *t*=0 during data processing. The raw data, which comprised 4,800 data points, were reduced to 500 points by averaging on a logarithmic time axis before analysis.

### Transient absorption data analysis and modelling

SVD-based rank analysis and global fitting were performed using an in-house written programme described previously^25^. Briefly, time-resolved data sets from transient absorption measurements are a series of difference spectra recorded at a number of delay times, Δ*A*(*t*, *λ*). For a set of discrete *N*_T_ time points *t*_*i*_ and *N*_L_ wavelengths *λ*_*j*_, these data form a rectangular matrix **ΔA** of dimension *N*_T_ × *N*_L_ with matrix elements Δ*A*_*ij*_=Δ*A*(*t*_*i*_, *λ*_*j*_). Each column of the matrix is a time trace for a fixed wavelength, each row is a spectrum at a given delay time. The data matrix can be decomposed into a sum of products of one-dimensional functions by a global fit:


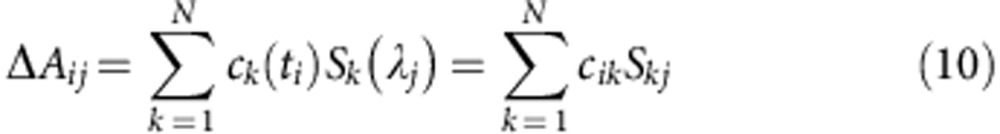


The global fit assumes that the data can be modelled by a factorization into a sum of products of spectra, *S*_*k*_(*λ*), and concentration time profiles, *c*_*k*_(*t*), as defined in equation (10), where the time profiles can be written as a linear combination of known analytic functions *f*_*j*_(*t*):


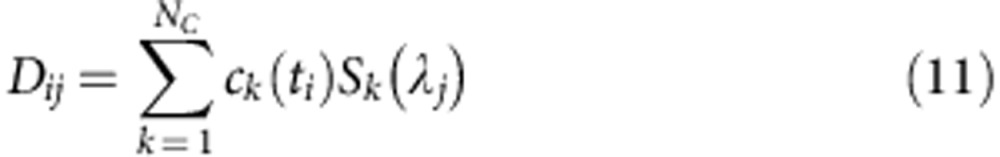



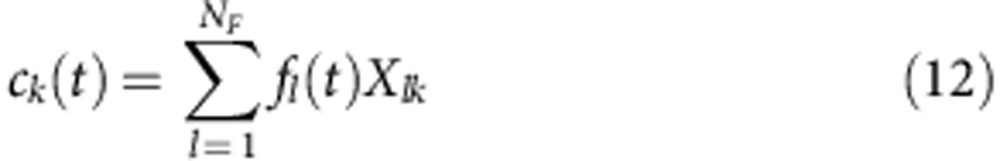


Here *N*_C_ is the number of distinct spectral species and *N*_F_ is the number of analytic time functions. Defining **C** as a *N*_T_ × *N*_C_ matrix with elements, *C*_*ik*_=*c*_*k*_(*t*_*i*_), and **F** as a *N*_T_ × *N*_C_ matrix with elements, *F*_*il*_=*f*_*l*_(*t*_*i*_), the model data matrix **D** can be written as:


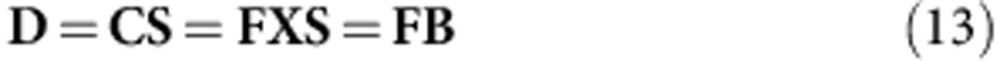


The *k*-th row of the matrix **B**, with elements *B*_*kj*_=*b*_*k*_(*λ*_*j*_), corresponds to the spectral changes associated with the time function, *f*_*k*_(*t*). When these time functions are exponential decays (convoluted with the instrument response), the corresponding spectra are called DADS. Optimization includes solving the least squares optimization problem:


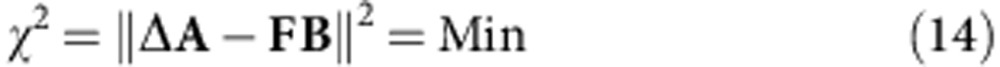


Efficient algorithms exist that solve equation (14) for fixed matrices **ΔA** and **F**. The value of *χ*^2^ optimized in this way is further minimized by a nonlinear least squares algorithm by variation of the rate constants in **F**. The DADS and the corresponding rate constants are the unique result of this global fit. This treatment does not require any model for the kinetics involved in the transient processes. The number of exponentials to use in the global fit is determined by SVD-based rank analysis, which is described in detail in the Supplementary Information of Lanzl *et al.*^35^. The details of a model will be entirely defined in the matrix **X** that relates the actual species kinetics to the elementary function, *f*_*k*_(*t*). The appropriate matrix, **X**, was chosen based on the model in Fig. 5a, and the SAS in matrix **S** was calculated by:


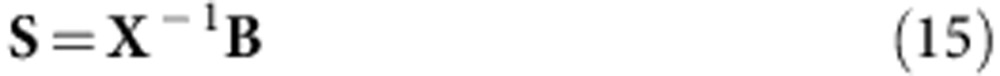


We tested the validity of each mechanistic model in two steps (see Supplementary Figs 18–21 and Supplementary Methods for full details): (i) the unique DADS are determined by the global lifetime analysis described above; (ii) various kinetic schemes are projected onto these DADS according to equation (15). Since the second step does not change the *χ*^2^ value found in the first step, this procedure has the advantage that all interpretation is performed with the same quality of fit.

## Additional information

**How to cite this article:** Kutta, R. J. *et al.* The photochemical mechanism of a B_12_-dependent photoreceptor protein. *Nat. Commun.* 6:7907 doi: 10.1038/ncomms8907 (2015).

## Supplementary Material

Supplementary Information Supplementary Figures 1-21, Supplementary Methods and Supplementary References

## References

[b1] Brown K. L. Chemistry and enzymology of vitamin B_12_. Chem. Rev. 105, 2075–2149 (2005) . 15941210 10.1021/cr030720z

[b2] Reed G. H. Radical mechanisms in adenosylcobalamine-dependent enzymes. Curr. Opin. Chem. Biol. 8, 477–483 (2004) . 15450489 10.1016/j.cbpa.2004.08.008

[b3] Johnson J. E. Jr, Reyes F. E., Polaski J. T. & Batey R. T. B_12_ cofactors directly stabilize an mRNA regulatory switch. Nature 492, 133–137 (2012) . 23064232 10.1038/nature11607 PMC3518761

[b4] Peselis A. & Serganov A. Structural insights into ligand binding and gene expression control by an adenosylcobalamin riboswitch. Nat. Struct. Mol. Biol. 19, 1182–1184 (2012) . 23064646 10.1038/nsmb.2405

[b5] Ortiz-Guerrero J. M., Polanco M. C., Murillo F. J., Padmanabhan S. & Elías-Arnanz M. Light-dependent gene regulation by a coenzyme B_12_-based photoreceptor. Proc. Natl Acad. Sci. USA 108, 7565–7570 (2011) . 21502508 10.1073/pnas.1018972108 PMC3088613

[b6] Weissbach H., Ladd J. N., Volcani B. E., Smyth R. D. & Barker H. A. Structure of the adenylcobamide coenzyme: degradation by cyanide, acid, and light. J. Biol. Chem. 235, 1462–1473 (1960) . 13843764

[b7] Pratt J. M. The chemistry of vitamin B_12_. Part II. Photochemical reactions. J. Chem. Soc. 5154–5160 (1964) .

[b8] Jones A. R., Levy C., Hay S. & Scrutton N. S. Relating localized protein motions to the reaction coordinate in coenzyme B_12_-dependent enzymes. FEBS J. 280, 2997–3008 (2013) . 23462350 10.1111/febs.12223

[b9] Armstrong G. A. Genetics of eubacterial carotenoid biosynthesis: a colorful tale. Annu. Rev. Microbiol. 51, 629–659 (1997) . 9343362 10.1146/annurev.micro.51.1.629

[b10] Cervantes M. & Murillo F. J. Role for vitamin B_12_ in light induction of gene expression in the bacterium *Myxococcus xanthus*. J. Bacteriol. 184, 2215–2224 (2002) . 11914353 10.1128/JB.184.8.2215-2224.2002 PMC134944

[b11] Pérez-Marín M. C., Padmanabhan S., Polanco M. C., Murillo F. J. & Elías-Arnanz M. Vitamin B_12_ partners the CarH repressor to downregulate a photoinducible promoter in *Myxococcus xanthus*. Mol. Microbiol. 67, 804–819 (2008) . 18315685 10.1111/j.1365-2958.2007.06086.x

[b12] Díez A. *et al.* Analytical ultracentrifugation studies of oligomerization and DNA-binding of TtCarH, a *Thermus thermophilus* coenzyme B_12_-based photosensory regulator. Eur. Biophys. J. 42, 463–476 (2013) . 23512413 10.1007/s00249-013-0897-x

[b13] Cheng Z., Li K., Hammad L. A., Karty J. A. & Bauer C. E. Vitamin B_12_ regulates photosystem gene expression via the CrtJ antirepressor AerR in *Rhodobacter capsulatus*. Mol. Microbiol. 91, 649–664 (2014) . 24329562 10.1111/mmi.12491 PMC3946051

[b14] Marques H. M., Marsh J. H., Mellor J. R. & Munro O. Q. The coordination of imidazole and its derivatives by aquocobalamin. Inorg. Chim. Acta 170, 259–269 (1990) .

[b15] Rury A. S., Wiley T. E. & Sension R. J. Energy cascades, excited state dynamics, and photochemistry in cob(III)alamins and ferric porphyrins. Acc. Chem. Res. 48, 860–867 (2015) . 25741574 10.1021/ar5004016

[b16] Shiang J. J. *et al.* Ultrafast excited-state dynamics in vitamin B_12_ and related cob(III)alamins. J. Am. Chem. Soc. 128, 801–808 (2006) . 16417369 10.1021/ja054374+

[b17] Walker L. A., Shiang J. J., Anderson N. A., Pullen S. H. & Sension R. J. Time-resolved spectroscopic studies of B_12_ coenzymes: the photolysis and geminate recombination of adenosylcobalamin. J. Am. Chem. Soc. 120, 7286–7292 (1998) .

[b18] Yoder L. M., Cole A. G., Walker L. A. & Sension R. J. Time-resolved spectroscopic studies of B_12_ coenzymes: influence of solvent on the photolysis of adenosylcobalamin. J. Phys. Chem. B 105, 12180–12188 (2001) .

[b19] Jones A. R. *et al.* Ultrafast infrared spectral fingerprints of vitamin B_12_ and related cobalamins. J. Phys. Chem. A 116, 5586–5594 (2012) . 22612868 10.1021/jp304594d

[b20] Sension R. J. *et al.* Photolysis and recombination of adenosylcobalamin bound to glutamate mutase. J. Am. Chem. Soc. 126, 1598–1599 (2004) . 14871067 10.1021/ja0396910

[b21] Sension R. J. *et al.* Time-resolved measurements of the photolysis and recombination of adenosylcobalamin bound to glutamate mutase. J. Phys. Chem. B 109, 18146–18152 (2005) . 16853330 10.1021/jp052492d

[b22] Jones A. R., Hardman S. J. O., Hay S. & Scrutton N. S. Is there a dynamic protein contribution to the substrate trigger in coenzyme B_12_-dependent ethanolamine ammonia lyase? Angew. Chem. Int. Ed. 50, 10843–10846 (2011) . 10.1002/anie.201105132 21948289

[b23] Walker L. A. *et al.* Time-resolved spectroscopic studies of B_12_ coenzymes: the identification of a metastable cob(III)alamin photoproduct in the photolysis of methylcobalamin. J. Am. Chem. Soc. 120, 3597–3603 (1998) .

[b24] Robertson W. D. & Warncke K. Photolysis of adenosylcobalamin and radical pair recombination in ethanolamine ammonia-lyase probed on the micro- to millisecond time scale by using time-resolved optical absorption spectroscopy. Biochemistry 48, 140–147 (2009) . 19072291 10.1021/bi801659e PMC2642536

[b25] Kutta R.-J., Langenbacher T., Kensy U. & Dick B. Setup and performance of a streak camera apparatus for transient absorption measurements in the ns to ms range. Appl. Phys. B 111, 203–216 (2013) .

[b26] Chen E. & Chance M. R. Nanosecond transient absorption spectroscopy of coenzyme B_12_. Quantum yields and spectral dynamics. J. Biol. Chem. 265, 12987–12994 (1990) . 2376584

[b27] Chagovetz A. M. & Grissom C. B. Magnetic Field Effects in adenosylcob(III)alamin photolysis: relevance to B_12_ enzymes. J. Am. Chem. Soc. 115, 12152–12157 (1993) .

[b28] Jones A. R., Woodward J. R. & Scrutton N. S. Continuous wave photolysis magnetic field effect investigations with free and protein-bound alkylcobalamins. J. Am. Chem. Soc. 131, 17246–17253 (2009) . 19899795 10.1021/ja9059238

[b29] Chen Z.-G. *et al.* Dynamic, electrostatic model for the generation and control of high-energy radical intermediates by a coenzyme B_12_-dependent enzyme. ChemBioChem 14, 1529–1533 (2013) . 23959797 10.1002/cbic.201300420 PMC4155860

[b30] Jost M., Simpson J. H. & Drennan C. L. The transcription factor carh safeguards use of adenosylcobalamin as a light sensor by altering the photolysis products. Biochemistry 54, 3231–3234 (2015) . 25966286 10.1021/acs.biochem.5b00416 PMC4455981

[b31] Schrauzer G. N. & Holland R. J. Hydridocobalamin and a new synthesis of organocobalt derivatives of vitamin B_12_. J. Am. Chem. Soc. 93, 4060–4062 (1971) . 5138307 10.1021/ja00745a048

[b32] Garr C. D. & Finke R. G. Radical cage effects in adocobinamide (axial-base-off coenzyme B_12_): a simple method for trapping [Ado· ·Co^II^] radical pairs. A new *β*-H elimination product from the radical pair and measurement of an unprecedentedly large cage-recombination efficiency factor, *F*_c_≥0.94. J. Am. Chem. Soc. 114, 10440–10445 (1992) .

[b33] Garr C. D. & Finke R. G. Adocobalamin (AdoCbl or coenzyme B12) cobalt-carbon bond homolysis radical-cage effects: product, kinetic, mechanistic, and cage efficiency factor (Fc) studies, plus the possibility that coenzyme B12-dependent enzymes function as “ultimate radical cages” and “ultimate radical traps”. Inorg. Chem. 32, 4414–4421 (1993) .

[b34] Warren M. J., Raux E., Schubert H. L. & Escalante-Semerena J. C. The biosynthesis of adenosylcobalamin (vitamin B_12_). Nat. Prod. Rep. 19, 390–412 (2002) . 12195810 10.1039/b108967f

[b35] Lanzl K., Sanden-Flohe M. v., Kutta R.-J. & Dick B. Photoreaction of mutated LOV photoreceptor domains from *Chlamydomonas reinhardtii* with aliphatic mercaptans: implications for the mechanism of wild type LOV. Phys. Chem. Chem. Phys. 12, 6594–6604 (2010) . 20448867 10.1039/b922408d

[b36] Šali A. & Blundell T. L. Comparative protein modelling by satisfaction of spatial restraints. J. Mol. Biol. 234, 779–815 (1993) . 8254673 10.1006/jmbi.1993.1626

[b37] Drennan C. L., Huang S., Drummond J. T., Matthews R. G. & Ludwig M. L. How a protein binds B_12_: A 3.0 Å X-ray structure of B_12_-binding domains of methionine synthase. Science 266, 1669–1674 (1994) . 7992050 10.1126/science.7992050

[b38] Higgins D. G. & Sharp P. M. CLUSTAL: a package for performing multiple sequence alignment on a microcomputer. Gene 73, 237–244 (1988) . 3243435 10.1016/0378-1119(88)90330-7

[b39] Thompson J. D., Gibson T. J., Plewniak F., Jeanmougin F. & Higgins D. G. The CLUSTAL_X Windows interface: flexible strategies for multiple sequence alignment aided by quality analysis tools. Nucleic Acids Res. 25, 4876–4882 (1997) . 9396791 10.1093/nar/25.24.4876 PMC147148

[b40] Jost M. *et al.* Structural basis for gene regulation by a B_12_-dependent photoreceptor. Nature doi: 10.1038/nature14950 (2015) . 10.1038/nature14950 PMC4634937 26416754

