# Supplementary material for: The photochemical mechanism of a B12-dependent photoreceptor protein
Source: Nat Commun. 2015 Aug 12;6:7907. doi: 10.1038/ncomms8907 (PMC4557120; doi:10.1038/ncomms8907)
Supplement: Supplementary Information — Supplementary Figures 1-21, Supplementary Methods and Supplementary References [file ncomms8907-s1.pdf]

Supplementary Figures

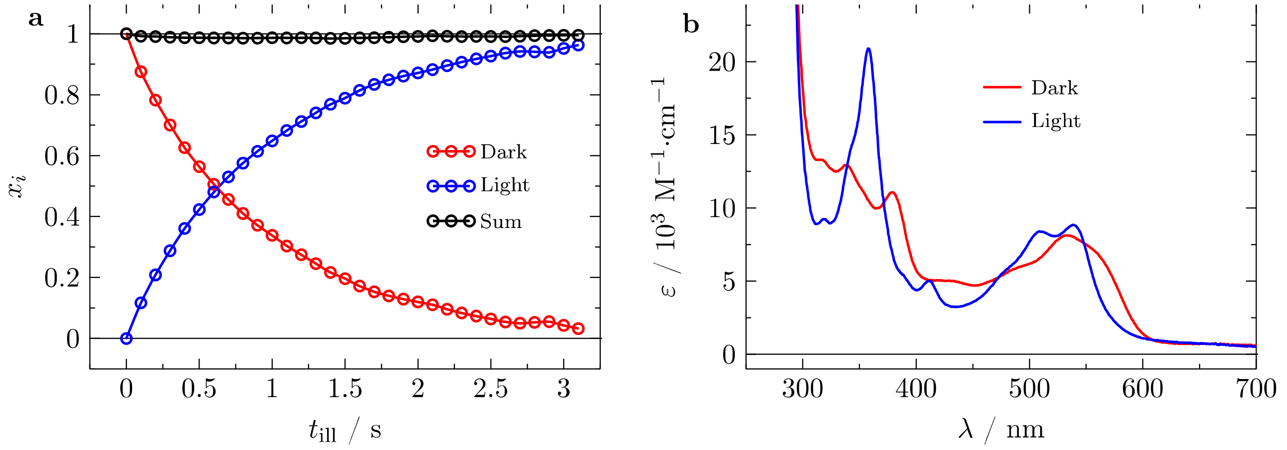

Supplementary Figure 1. Photoconversion of CarH-GS to CarH-LS. Deconvolution of the sequential spectra acquired after stepwise illumination (λ_{max} = 530 nm) of CarH-GS. **a.** Mole fraction (x_i) *vs.* illumination time (t_{ill}) profiles of CarH-GS (red), CarH-LS (blue) and their sum (black). **b.** Pure species spectra of CarH-GS (red) and CarH-LS (blue).

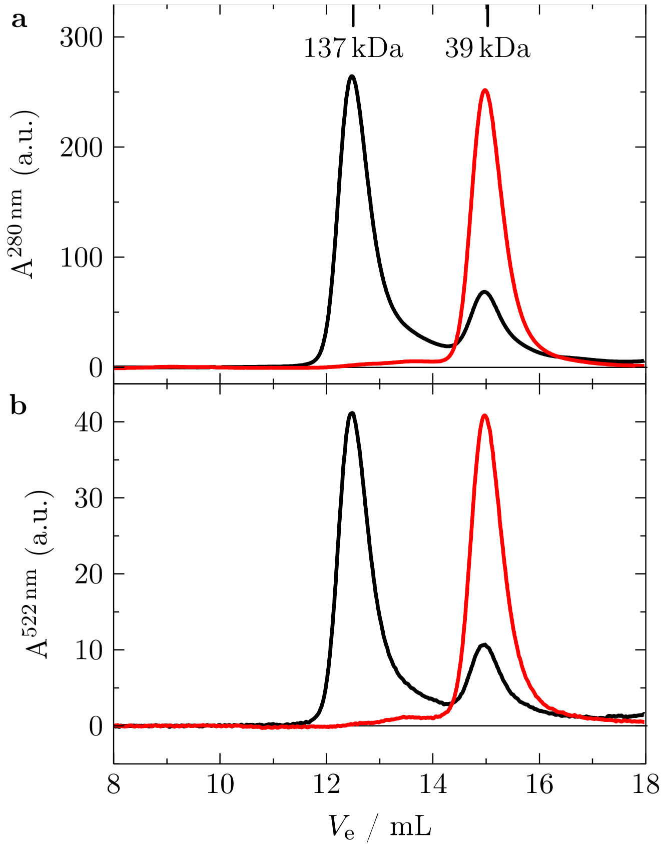

Supplementary Figure 2. Size Exclusion Chromatography. Traces recorded at 280 nm (**a**) and 522 nm (**b**) of the elution from size exclusion chromatography. Black traces represent OHCbl-bound CarH, and the red traces CarH-LS, both pre-incubated *in vitro* with a five-fold excess of AdoCbl. The black traces show a reversible formation of the CarH-GS tetramer (137 kDa), which means that AdoCbl is able to displace OHCbl (previously shown to bind to CarH).[1] The red traces show only the CarH-LS monomer (39 kDa), which means after photoconversion *in vitro*, exogenous AdoCbl cannot displace the cobalamin bound to CarH-LS.

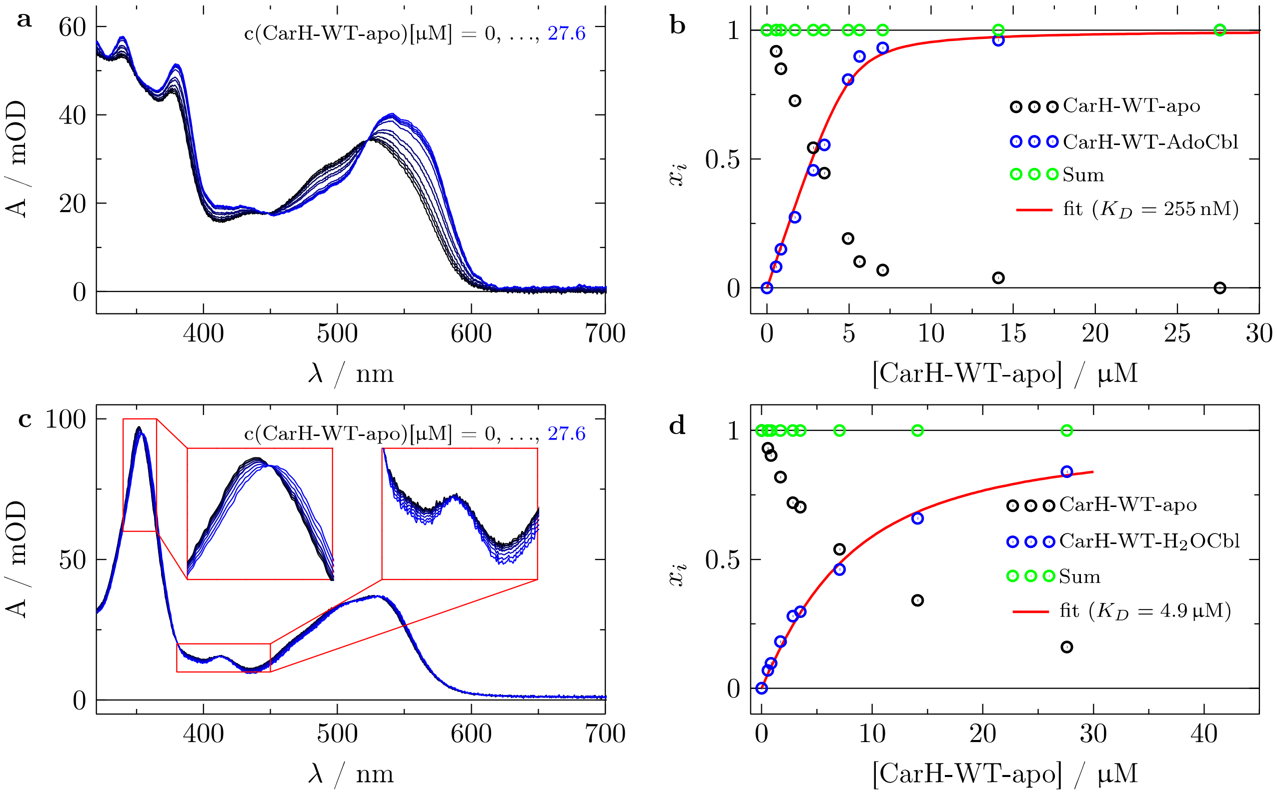

Supplementary Figure 3. Spectral determination of cobalamin binding affinities to CarH. Spectral evolution and corresponding mole fraction profiles of AdoCbl (**a** and **b**) and OHCbl (**c** and **d**) during titration with apoCarH. In **a** and **c**, low concentrations of CarH – black lines; higher concentrations of CarH – blue lines. The red lines in **b** and **d** are the least square fits according to eq. 5 of the main article.

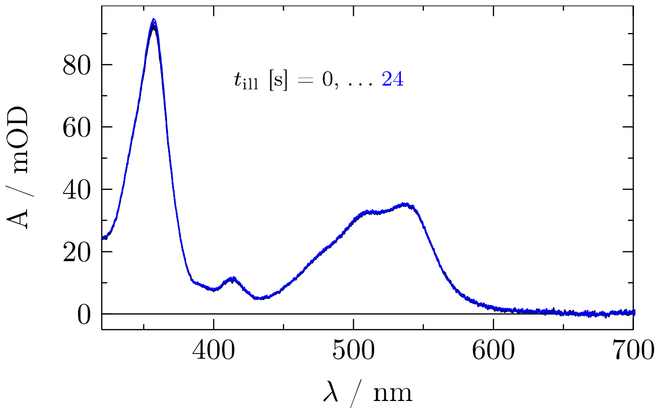

Supplementary Figure 4. Illumination of CarH-bound OHCbl. UV-visible spectra of CarH-bound OHCbl (or, at pH 7.5, H_2OCbl) acquired after stepwise illumination with a pulsed LED (λ_{max} = 530 nm) for up to 24 s (black-blue). These data are consistent with the CarH-LS adduct, which has significantly more pronounced structure on the peaks between 500 – 600 nm (Figure 1a of the main manuscript), not forming under illumination of OHCbl / H_2OCbl bound to CarH on this timescale.

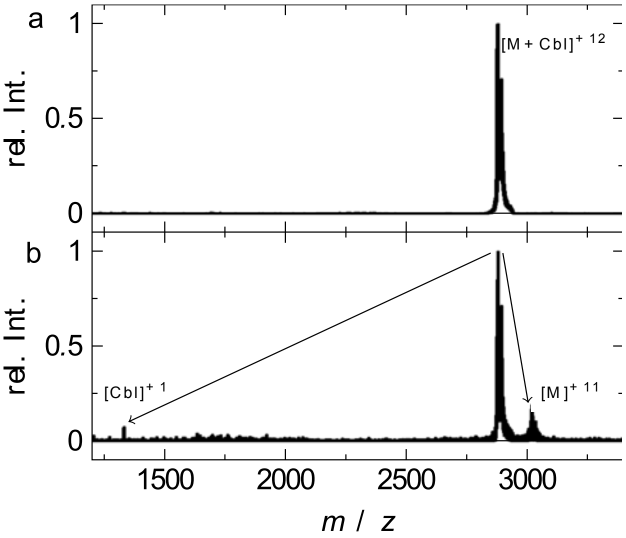

Supplementary Figure 5. Collision-induced dissociation mass spectrometry. a. Mass selection of the $[M+Cbl]^{12+}$ complex (2,873 m/z) using the quadrupole of a Synapt G2 mass spectrometer. When no collision energy is applied we only observe the mass distribution of the precursor ion ($[M+Cbl]^{12+}$ complex). **b.** Collision of the sample with argon in the trap cell region results in fragmentation into two additional ions: a mass, M^{11+} (3,014 m/z), corresponding to that of the CarH apo-monomer, and Cbl^{+} (cobalamin, 1,329 m/z). This is clear evidence of binding between the protein and Cbl following photoexcitation of CarH-GS.

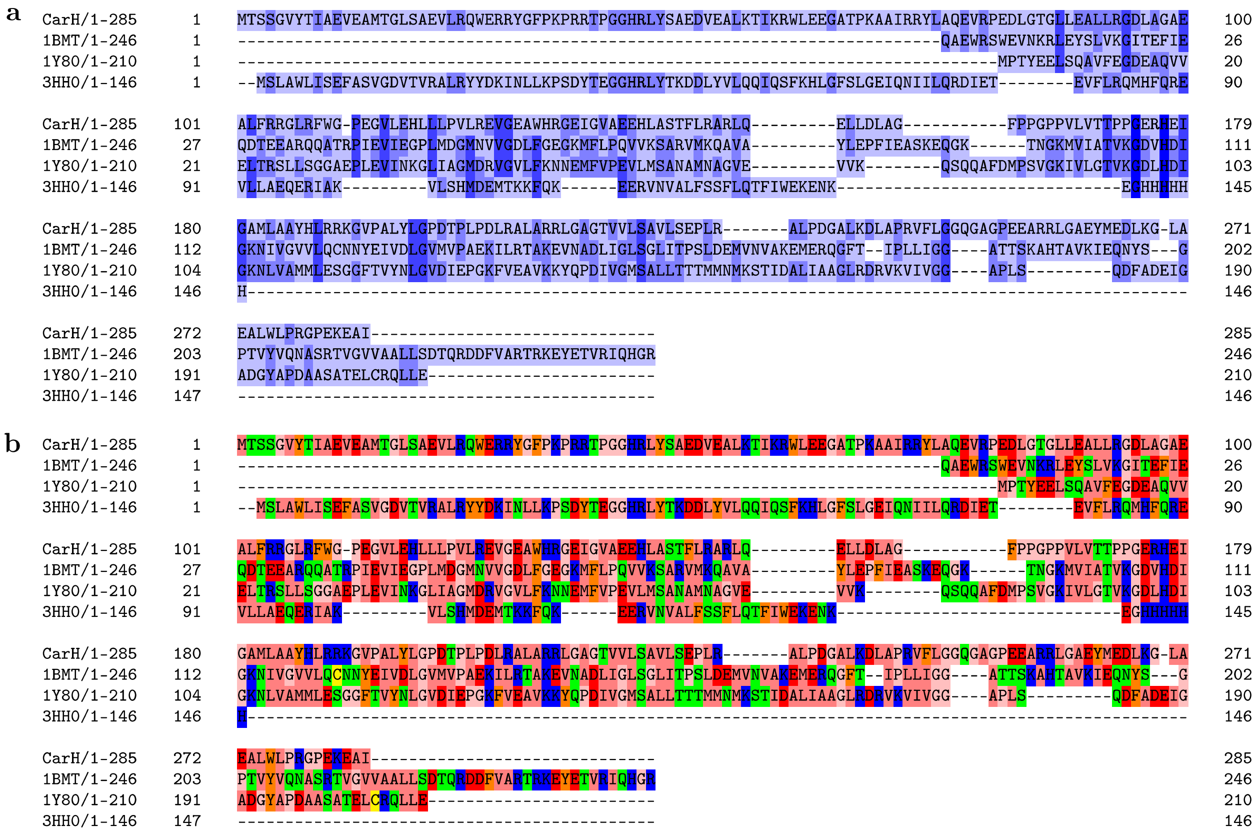

Supplementary Figure 6. Sequence alignment of the CarH homology model templates. The full length CarH target sequence was aligned with the sequences from three template structures: methionine synthase from *E. coli* (pdb code: 1BMT, dimeric); a corrinoid (factor IIIm)-binding protein from *Moorella thermoacetica* (pdb code: 1Y80, monomeric); a transcriptional regulator of the MerR family from *Bacillus cereus* (pdb code: 3HH0, tetrameric). **a.** Darker blue backgrounds indicate identical amino acids in at least two sequences. **b.** Residues are coloured according to their physicochemical properties as follows: aliphatic/hydrophobic (ILVAM) pale rose; aromatic (FWY) orange; positively charged (KRH) blue; negatively charged (DE) red; hydrophilic (STNQ) green; conformationally special (PG) pink; cysteine (C) yellow.

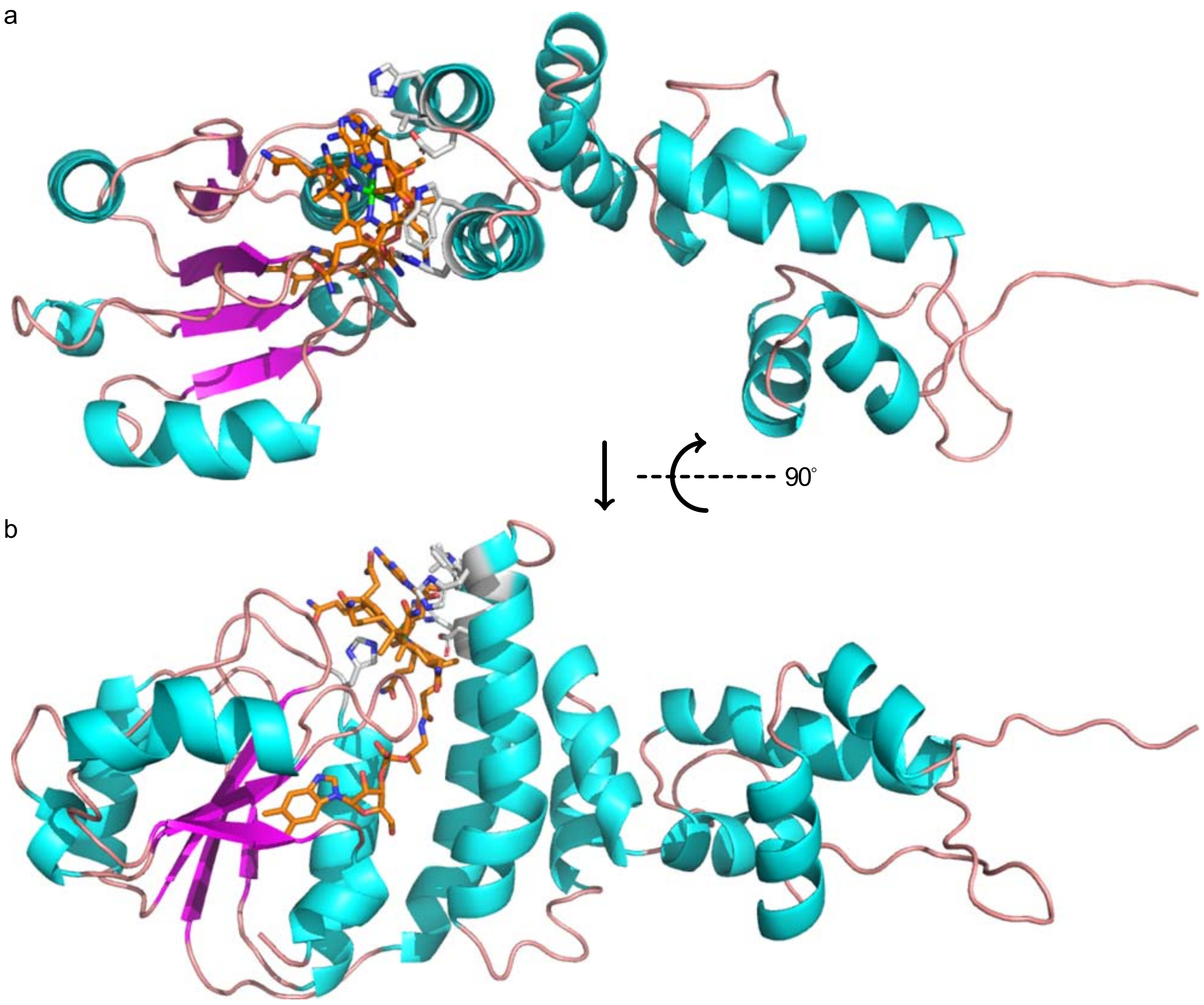

Supplementary Figure 7. Homology model structures of CarH. Overall CarH homology model monomer structure shown from the top face (**a**) and side (**b**) of AdoCbl (shown in orange). The model was based on the template structures: methionine synthase from *E. coli* (PDB code: 1BMT, dimeric),[2] corrinoid (factor IIIm)-binding protein from *Moorella thermoacetica* (PDB code: 1Y80, monomeric) and a transcriptional regulator of the MerR family from *Bacillus cereus* (PDB code: 3HH0, tetrameric). The overall monomer structure shows an elongated, aspheric form in good agreement with sedimentation velocity studies,[1] which indicate that, in solution, CarH-apo and light-exposed CarH-LS are predominantly ellipsoidal monomers. The N-terminal DNA binding (H-T-H motif) domain is on the right and the C-terminal AdoCbl binding domain is on the left. They are connected *via* an interaction between two α-helices on one and two α-helices on the other. The two helices on the AdoCbl binding domain are approximately twice as long as those on the DNA binding domain, and interact with the AdoCbl chromophore *via* the upper 5’-deoxyadenosyl ligand and the linker between the corrin ring and the 5,6-dimethylbenzimidazole moiety.

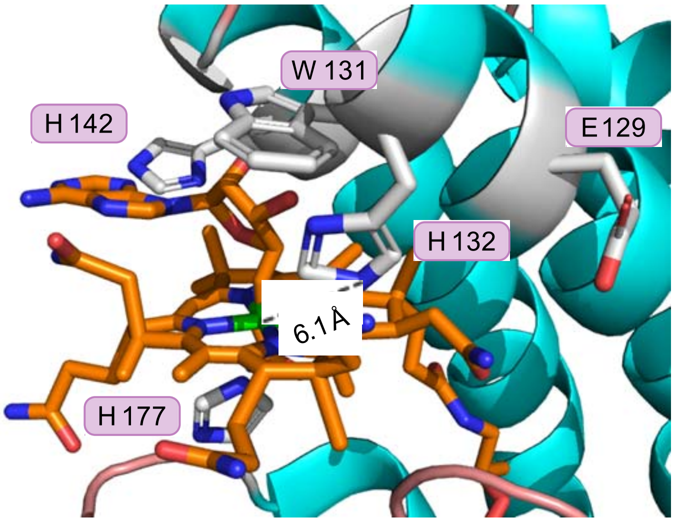

Supplementary Figure 8. AdoCbl-binding pocket from the homology model of CarH. The chromophore is bound ‘base off’, with the 5,6-dimethylbenzimidazole displaced by H177. A tryptophan (W131) residue occupies the space directly above the corrin ring. The template structures for the photoreceptor domain (1BMT and 1Y80) are bound to methylcobalamin. Therefore, the structure of the much more bulky, upper axial 5’-deoxyadenosyl ligand was manually rebuilt into the PDB file with minimised steric interactions with the surrounding amino acids, especially W131. This is only possible if the 5’-deoxyadenosyl points towards the solvent space. Two histidine residues (H132 and H142) are also adjacent to the upper face of AdoCbl, either of which might plausibly form a covalent linkage with the Co after photocleavage of the Co−C bond to the 5’-deoxyadenosyl. E129 is available to deprotonate H132 if required to facilitate nucleophilic attack.

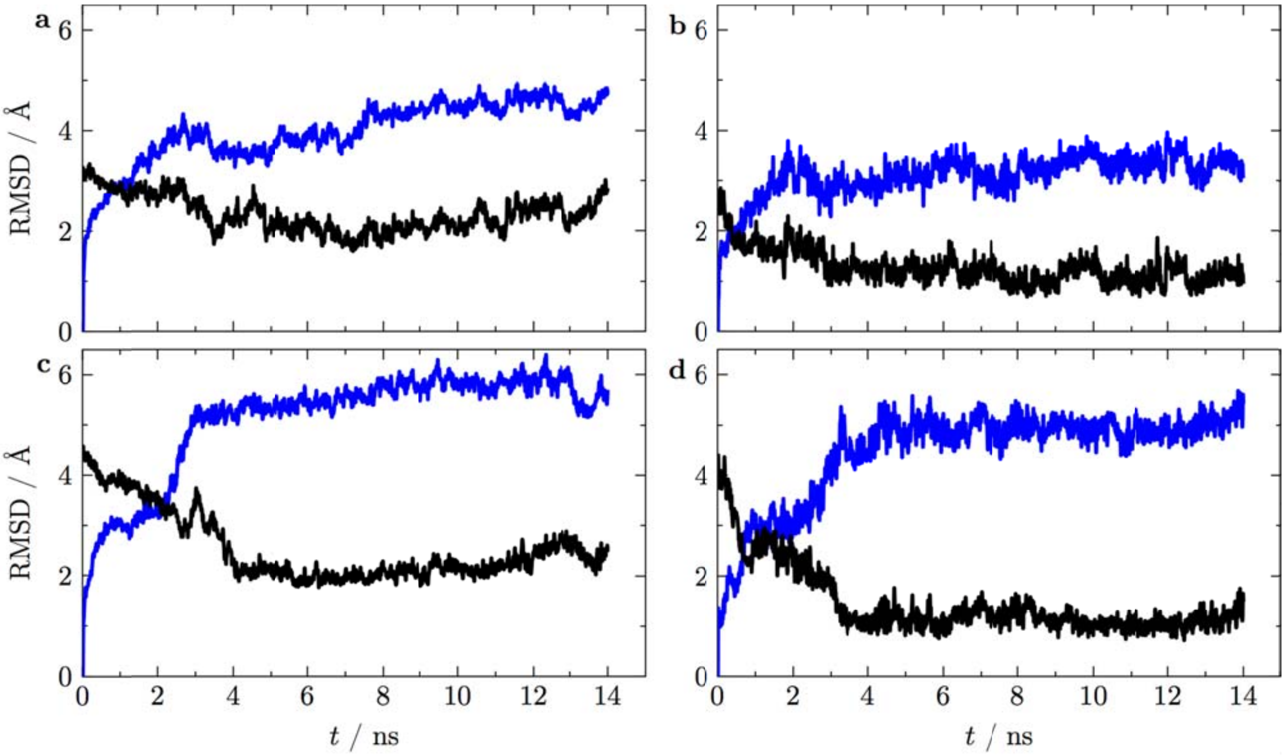

Supplementary Figure 9. Heavy-atom *root mean square deviation* plots. Molecular dynamics (MD) simulations were run for 10 ns following 4 ns of relaxation after alignment to the heavy atoms of the cobalamin-binding domain. Blue lines: *root mean square deviation* (RMSD) relative to the starting structure; black lines: RMSD relative to the average structure. **a.** RMSD of the cobalamin-binding domain with 5'-desoxyadenosyl bound; **b.** RMSD of cobalamin and H132 with 5'-desoxyadenosyl bound; **c.** RMSD of the cobalamin-binding domain without 5'-desoxyadenosyl bound; **d.** RMSD of cobalamin and H132 without 5'-desoxyadenosyl bound. 10 ns simulations proved sufficient to identify a major change in the relative orientation of the corrin ring. When 5'-deoxyadenosyl is bound, the RMSD of the AdoCbl-binding domain relative to the average structure (**a**) shows a slow drift in the conformation of the overall structure over the course of the simulation. The RMSD gently decreases towards the structure most similar to the average (near the middle of the simulation) then gently rises again. However, the cobalamin/H132 RMSD (**b**) shows that these moieties have reached a stable conformation after ~ 3 ns. When 5'-deoxyadenosyl is removed, significant structural changes occur in the first 3.5 ns (**c**), which decrease the distance between the Co central to the corrin and H132 (Supplementary Fig. 10).

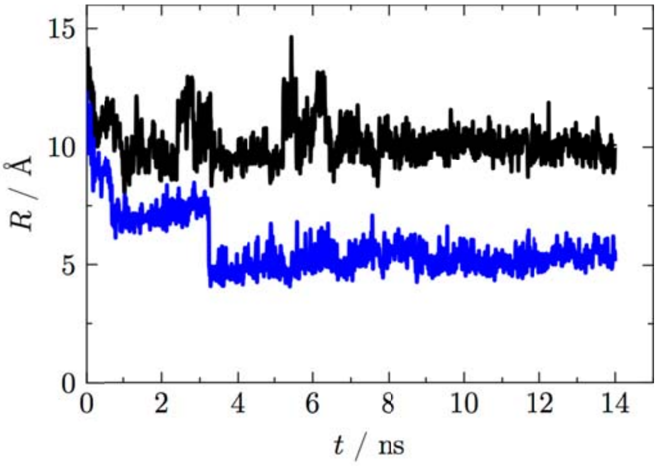

Supplementary Figure 10. Centre-of-mass distance between Co and H132 sidechain. Distances, *R*, were calculated between heavy atoms for MD simulations with 5'-desoxyadenosyl bound (black line) and without 5'-desoxyadenosyl (blue line). This represents a switch in conformation of the corrin ring from the starting structure (Figure 2a and b of the main article) to a position where H132 is directly above the Co (Figure 2c and d). Thus, after photolysis, H132 is ideally positioned to form an adduct with the Co and can be deprotonated by E129 to facilitate nucleophillic attack. That these changes start to occur so rapidly – almost as soon as the restraints are lifted during the 250 K phase – suggests that there is an insignificant barrier to this transition. The Co-H132 distance remains near 5 Å for the remaining 10 ns, indicating that these moieties have reached a stable relative conformation, even if the cobalamin/H132 conformation drifts slightly within the protein (Supplementary Figure 9d).

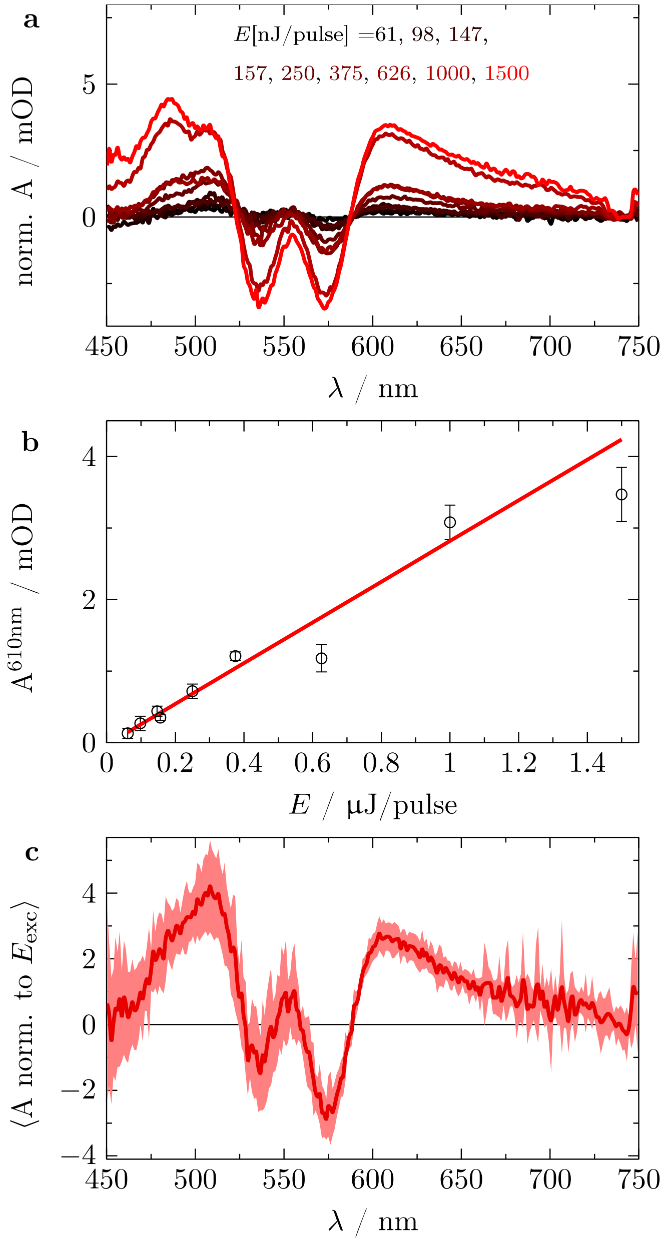

Supplementary Figure 11. Dependence on laser power of the 15 ps difference spetrum. a. Difference spectra amplitudes for the signal at 15 ps delay time (Figure 3b of the main article), corrected for the pre-t_0 signal, at increasing laser power (61 – 1500 nJ per pulse; black lines – low power; red lines – higher power). **b.** Absorbance change at 610 nm shows a linear trend as a function of laser power indicative of a single photon process. **c.** Average of all difference spectral normalised to their respective laser power (dark red) with the standard deviation (pale red) confirming linearity over the entire wavelength range.

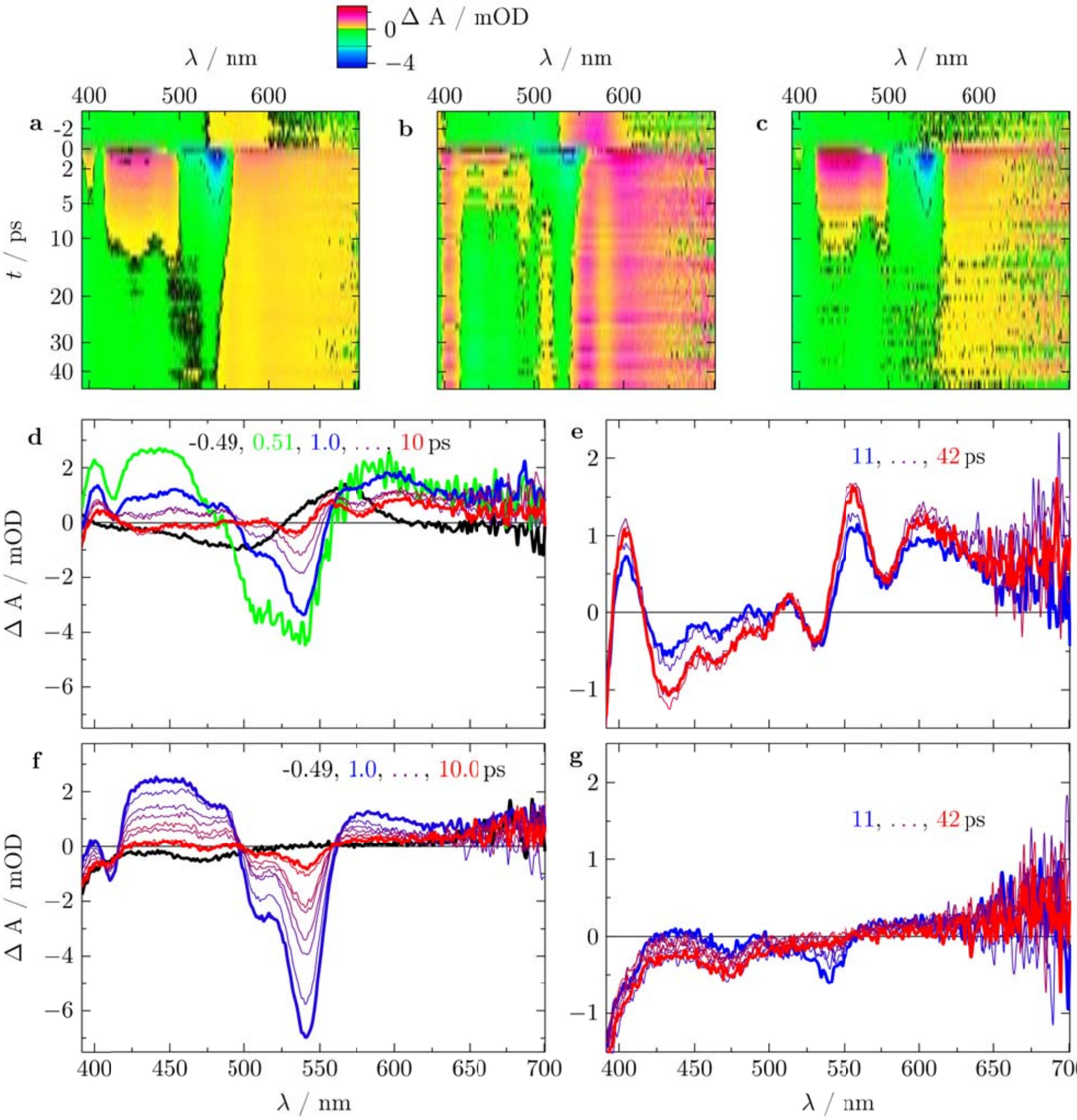

Supplementary Figure 12. CarH transient absorption data from 10 sequential scans. False colour representations (yellow to red – positive amplitudes; green to blue – negative amplitudes) of the data up to 50 ps delay time: averaged over all 10 scans (**a**); the first scan (**b**); the last scan (**c**). **d – e.** Example difference spectra from the first scan. **f – g.** Example difference spectra from the last scan. As the number of repeated scans increases the nature of the transient absorption signal changes. In contrast to the data from the first scan, the photodynamics in the last scan are complete within ~ 15 ps, after which only the baseline signal is remaining. These data further support the irreversible formation of the CarH-LS.

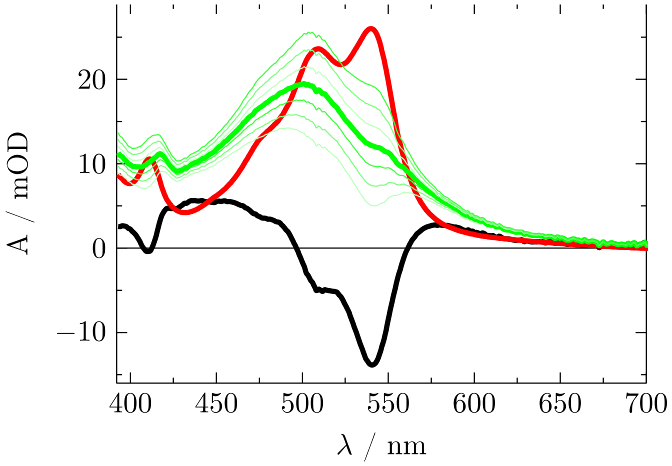

Supplementary Figure 13. Analysis of the ultrafast photoresponse of the CarH-LS. SVD and global analysis of the ultrafast transient absorption data representing the CarH-LS photoresponse gives a single decay constant, and therefore a simple 2 state model was applied. According to this model a reasonable species associated spectrum of the CarH-LS excited state (green) is generated by adding 5.5 % of the CarH-LS ground state spectrum (red) to the decay associated difference spectrum (DADS, black, lifetime: 3.2 ps) from the global analysis. Both the DADS and SAS have a high similarity with the excited state spectra of CNCbl.[3]

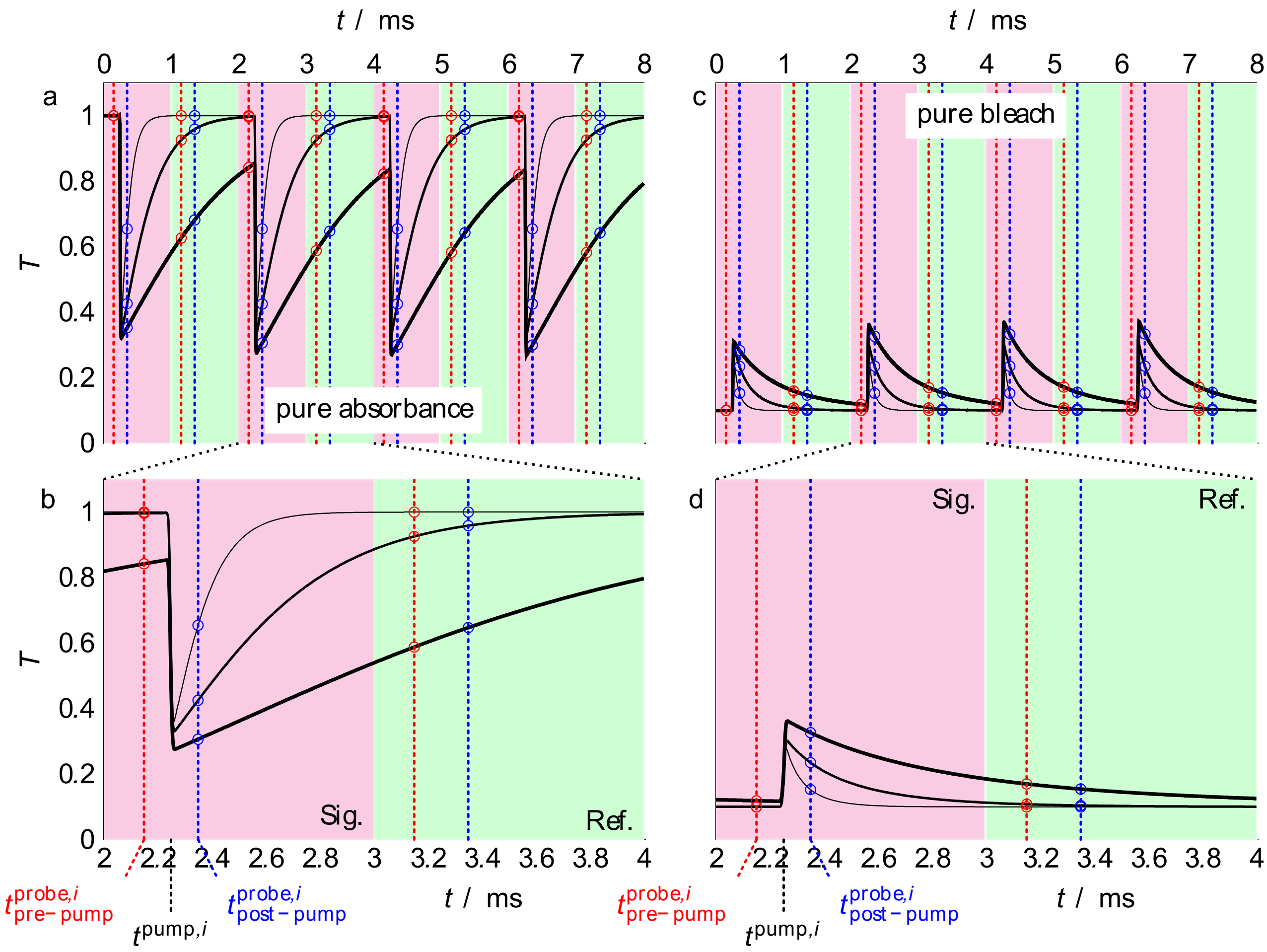

Supplementary Figure 14. Simulations of pre-t_0 pump probe transient absorption signals. The hypothetical transient absorption experiment runs at 0.5 kHz for the acquisition of a single pair of signal (pink area) and reference (green area) datasets. For simplicity, it is assumed that the sample features only a ground and excited state, and the sample consists of wavelengths at which the time evolution of the transmission arises only due to either pure absorbance of a new species (**a:** four subsequent pump-probe cycles shown with an expanded example in **b**) or pure bleach of the ground state absorbance (**c** and **d**). Three simulations for three different rate constants k_1 = 10 (ms)$^{-1}$, k_2 = 3 (ms)$^{-1}$ and k_3 = 1 (ms)$^{-1}$ for the decay of the excited state are shown in each. The circles indicate two individual probe pulses before and after the pump pulse. See also the methods section of the main article.

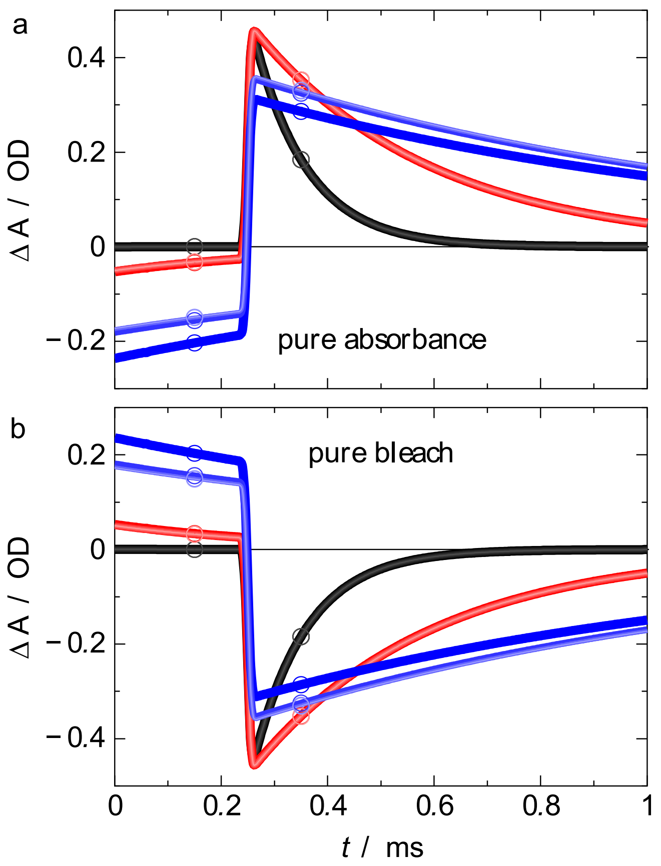

Supplementary Figure 15. Calculated transient absorption time profiles. The profiles for a pure absorption signal (**a**) and signal bleach (**b**) result from the simulations depicted in Supplementary Fig. 14. Three time profiles were calculated for k_1 = 10 $(ms)^{-1}$, (black), k_2 = 3 $(ms)^{-1}$ (red) and k_3 = 1 $(ms)^{-1}$ (blue). The four individual signal and reference datasets are overlaid for each time profile from dark to lighter tones. They show that species with lifetimes longer than the time difference between two sequential probe pulses result in an inverse transient absorption signal prior to the subsequent laser excitation. These signals also overlay with the transient absorption signals that evolve after the subsequent excitation pulse. The time window covered by the pump-probe experiment is typically two orders of magnitude smaller than the lifetime of the long-lived species. These contributions therefore manifest as a constant component in each data acquisition window. Therefore, it is necessary to use a constant function over all acquisition wavelengths in the global fit analysis. See also the methods section of the main article.

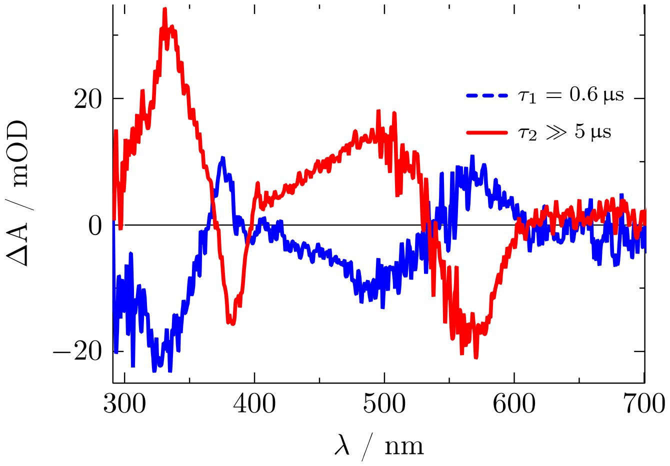

Supplementary Figure 16. DADS from for CarH-GS data acquired between ns – µs. The DADS result from SVD and global analysis of transient absorption described in the methods section of the main article**.** The blue line represents the DADS with lifetime, τ_1 = 0.6 µs, and the red line the DADS with lifetime, $\tau_2 \gg 5$ µs. However, the red DADS is equivalent to the pre-laser signal (*i.e.* the constant component, black DADs in Figure 4a) from the ultrafast data, and therefore has a lifetime > 1 ms.

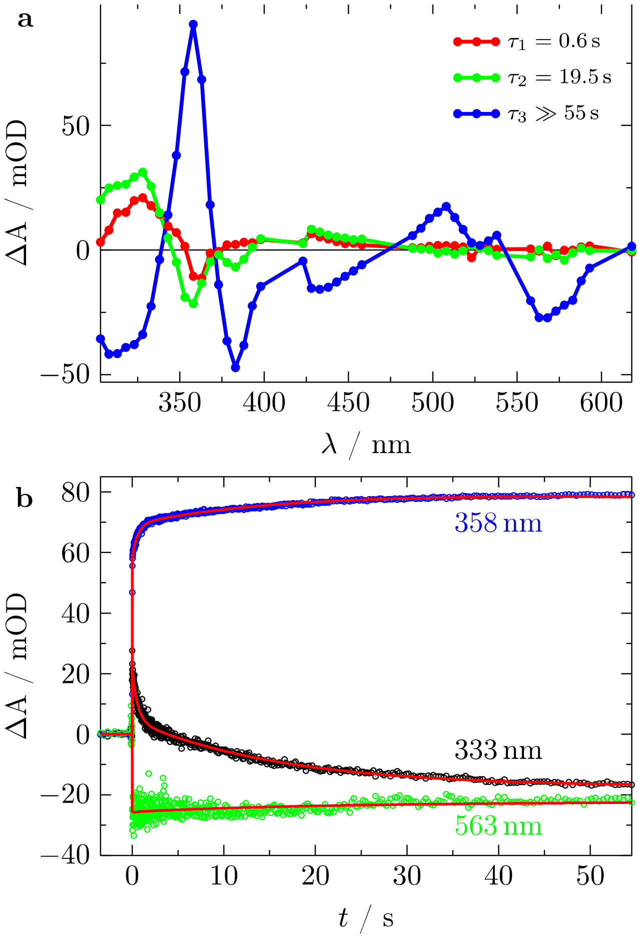

Supplementary Figure 17. Analysis of the ms – s CarH-GS transient absorption data. Data were acquired 12.5 ms to 60 s after the photoexcitation of CarH-GS. **a.** DADS from SVD-based rank analysis and global fitting, with lifetimes, τ_1 = 0.6 ms (red), τ_2 = 19.5 s (green) and $\tau_3 \gg$ 55 s. **b.** Temporal slices at 333 (black dots), 358 (blue dots) and 563 nm (green dots) plus corresponding global fits with three exponentials (red lines).

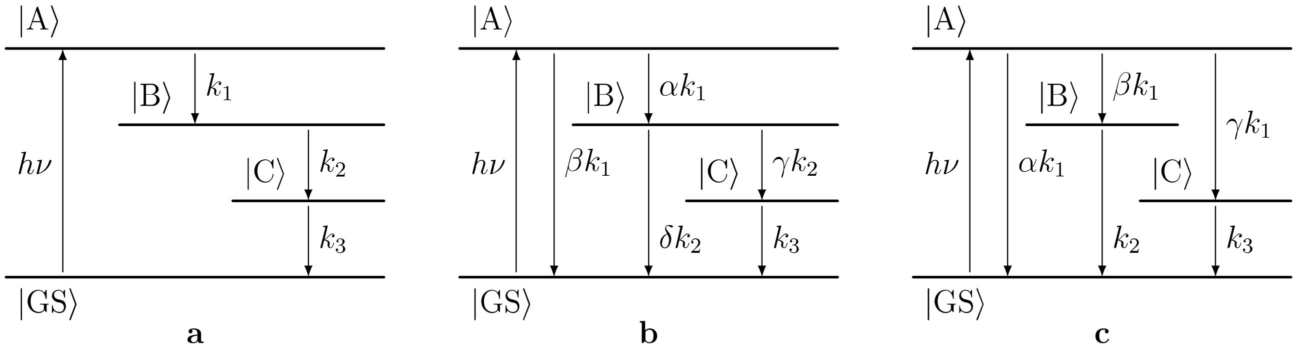

Supplementary Figure 18. Candidate models for the ultrafast photoresponse of CarH. The three kinetic models tested in order to describe the transient absorption data up to 3 ns following the photoexcitation of CarH-GS (Figure 3a of the main article). Each model comprises one ground state, |GS⟩, one excited state, |A⟩, and two intermediates, |B⟩ and |C⟩. **a.** A purely sequential model, **0**. **b.** A branched model, **1**, where branching ratios, $\alpha + \beta = 1$ and $\gamma + \delta = 1$. **c.** A branched model, **2**, where $\alpha + \beta + \gamma = 1$. Refer to Supplementary Figs. 19 – 21 and the Supplementary Methods section for a full discussion of each model and justification of which model best represents the data.

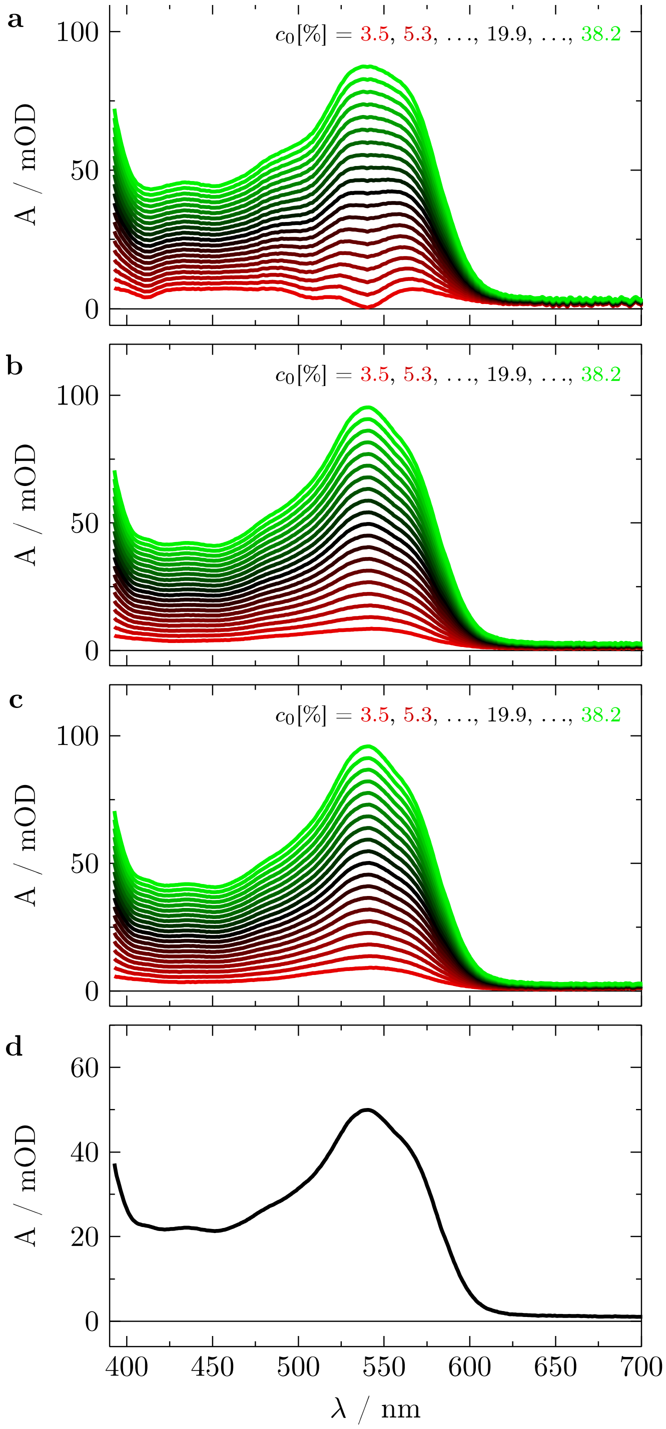

Supplementary Figure 19. Species associated spectra from model 0. SAS of |A⟩ (**a**), |B⟩ (**b**) and |C⟩ (**c**) from the ps – ns photodynamics of CarH-GS based on the purely sequential model **0** (Supplementary Fig. 18a). The fractional contribution, c_0, of the CarH-GS spectrum (**d**) was varied in **a – c** between 0.035 (red) and 0.382 (green). The preferred value of c_0 for species |A⟩, 0.199, results in almost identical SAS for species |B⟩ and |C⟩, (black lines, **a – c**).

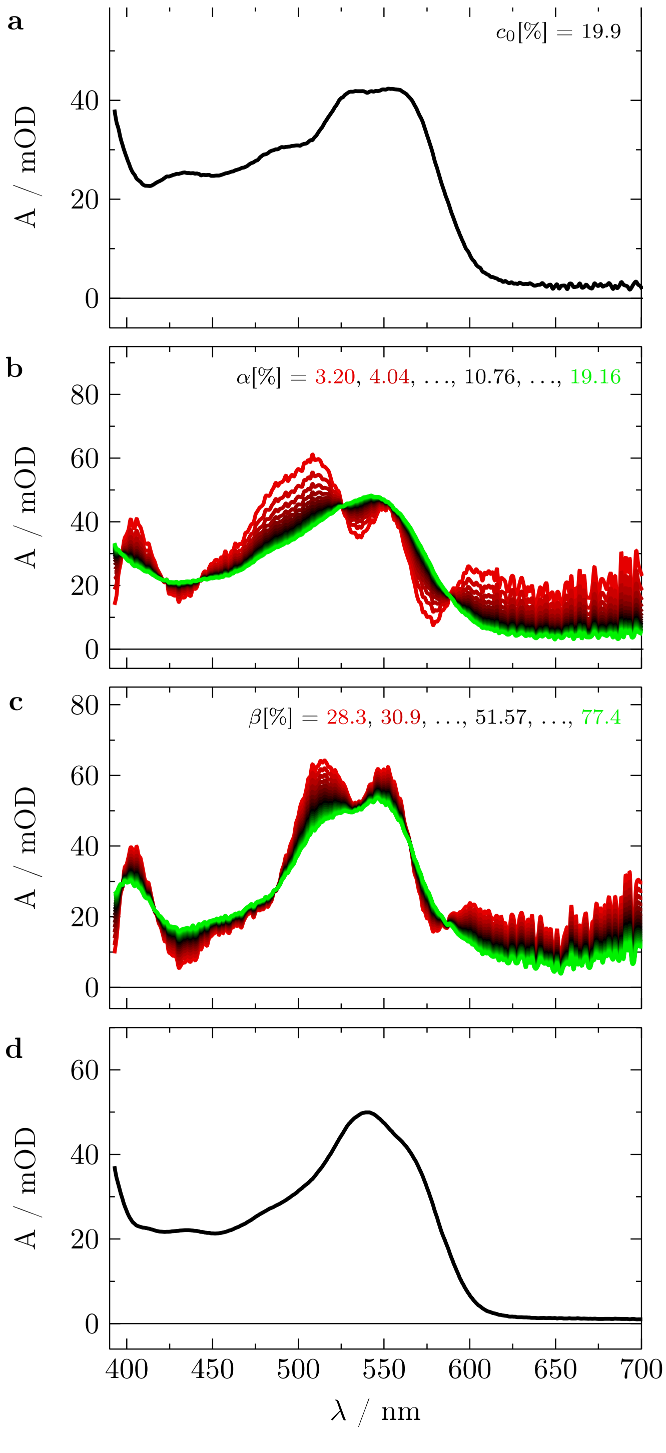

Supplementary Figure 20. Species associated spectra from model 1. SAS of |A⟩ (**a**), |B⟩ (**b**) and |C⟩ (**c**) from the ps – ns photodynamics of CarH-GS based on the branched model **1** (Supplementary Fig. 18b), where branching ratios, $\alpha + \beta = 1$ and $\gamma + \delta = 1$. The fractional contribution, c_0, of the CarH-GS spectrum (**d**) was kept constant in **a – c** at the value that determines the SAS of |A⟩, $c_0 = 0.199$. **b.** To determine the SAS of |B⟩ the branching ratio, α, was varied between 0.0320 (red) and 0.1916 (green) in 0.0084 steps, with the optimum spectrum achieved at $\alpha = 0.1076$ (black). **c.** To determine the SAS of |C⟩ the branching ratio, α, was fixed at 0.1076, and γ was varied between 0.283 (red) to 0.774 (green) in 0.026 steps, with the optimum spectrum achieved at $\gamma = 0.5157$ (black).

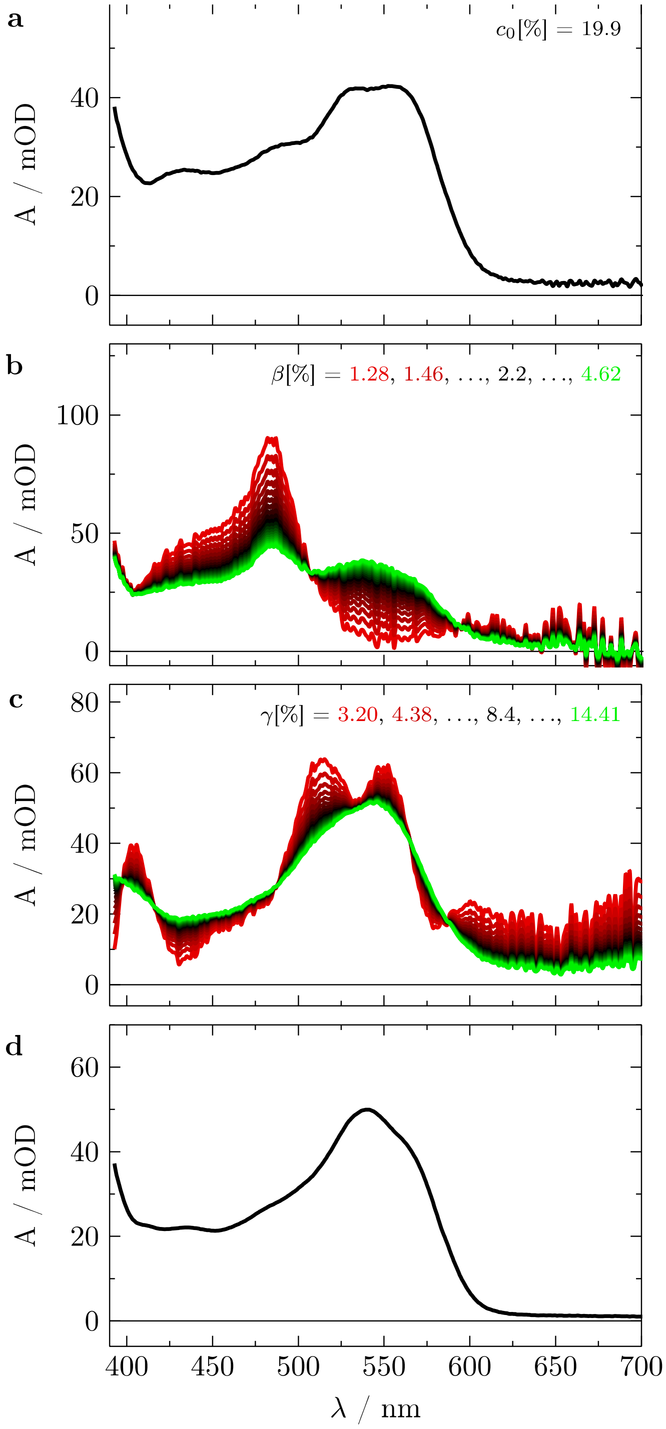

Supplementary Figure 21. Species associated spectra from model 2. SAS of |A⟩ (**a**), |B⟩ (**b**) and |C⟩ (**c**) from the ps – ns photodynamics of CarH-GS based on the branched model **2** (Supplementary Fig. 18c), where branching ratios, $\alpha + \beta + \gamma = 1$. The fractional contribution, c_0, of the CarH-GS spectrum (**d**) was kept constant in **a – c** at the value that determines the SAS of |A⟩, $c_0 = 0.199$. To determine the SAS of |B⟩ the branching ratio, β was varied between 0.0128 (red) and 0.0462 (green) in 0.0018 steps, with the optimum spectrum achieved at $\beta = 0.022$ (black). **c.** To determine the SAS of |C⟩ the branching ratio, γ, was varied between 0.0320 (red) to 0.1441 (green) in 0.00118 steps, with the optimum spectrum achieved at $\gamma = 0.084$ (black).

Supplementary Methods

Modelling of the ultrafast transient absorption data

Global fitting of transient absorption data results in DADS that then can be linearly combined based on a model to generate SAS. The fs – ns data following the photoexcitation of CarH-GS required the sum of three exponentials for a good quality fit. Therefore, only models that consist of a system of differential equations with three eigenvalues were applied. Three different models were used which consist of one ground state, $|\mathrm{GS}\rangle$, one excited state, $|\mathrm{A}\rangle$, and two intermediates, $|\mathrm{B}\rangle$ and $|\mathrm{C}\rangle$. A schematic drawing of each is shown in Supplementary Fig. 18.

Model **0** is a purely sequential model where $|\mathrm{GS}\rangle$ is excited into $|\mathrm{A}\rangle$, which converts completely into $|\mathrm{B}\rangle$, which in turn converts completely into $|\mathrm{C}\rangle$. Finally, $|\mathrm{C}\rangle$ decays back into $|\mathrm{GS}\rangle$, the rate constant for which is set to zero and corresponds to the long-lived DADS from the global fit. After excitation from the $|\mathrm{GS}\rangle$ into $|\mathrm{A}\rangle$, model 1 includes a branching from $|\mathrm{A}\rangle$ to $|\mathrm{GS}\rangle$ and to $|\mathrm{B}\rangle$, and $|\mathrm{B}\rangle$ then branches into $|\mathrm{GS}\rangle$ and $|\mathrm{C}\rangle$. Here, the rate constant of the decay of $|\mathrm{C}\rangle$ into $|\mathrm{GS}\rangle$ is zero as in model **0**. After excitation from the $|\mathrm{GS}\rangle$ into $|\mathrm{A}\rangle$ in Model 2, there is a three way branching of $|\mathrm{A}\rangle$ to $|\mathrm{GS}\rangle$, $|\mathrm{B}\rangle$, and $|\mathrm{C}\rangle$. Species $|\mathrm{B}\rangle$ and $|\mathrm{C}\rangle$ can then decay back into the ground state while the rate constant of the decay of $|\mathrm{C}\rangle$ is set to zero as in the other two models. Based on the individual models, the three DADS (D) corresponding to the three rate constants, k_1, k_2, and k_3, are then expressed in terms of the species associated difference spectra (SADS, S):

Model 0:

$$D_1 = S_{\mathrm{A}}^{(0)} - \frac{k_1}{k_1 - k_2} S_{\mathrm{B}}^{(0)} + \frac{k_1 k_2}{(k_1 - k_2)(k_1 - k_3)} S_{\mathrm{C}}^{(0)} \quad (1)$$

$$D_2 = \frac{k_1}{k_1 - k_2} S_{\mathrm{B}}^{(0)} - \frac{k_1 k_2}{(k_1 - k_2)(k_2 - k_3)} S_{\mathrm{C}}^{(0)} \quad (2)$$

$$D_3 = \frac{k_1 k_2}{(k_1 - k_3)(k_2 - k_3)} S_{\mathrm{C}}^{(0)} \quad (3)$$

Model 1:

$$D_1 = S_{\mathrm{A}}^{(1)} - \frac{\alpha k_1}{k_1 - k_2} S_{\mathrm{B}}^{(1)} + \frac{\alpha\gamma k_1 k_2}{(k_1 - k_2)(k_1 - k_3)} S_{\mathrm{C}}^{(1)} \quad (4)$$

$$D_2 = \frac{\alpha k_1}{k_1 - k_2} S_{\mathrm{B}}^{(1)} - \frac{\alpha\gamma k_1 k_2}{(k_1 - k_2)(k_2 - k_3)} S_{\mathrm{C}}^{(1)} \quad (5)$$

$$D_3 = \frac{\alpha\gamma k_1 k_2}{(k_1 - k_3)(k_2 - k_3)} S_C^{(1)} \tag{6}$$

Model 2:

$$D_1 = S_A^{(2)} - \frac{\alpha k_1}{k_1 - k_2} S_B^{(2)} - \frac{\beta k_1}{k_1 - k_3} S_C^{(2)} \tag{7}$$

$$D_2 = \frac{\alpha k_1}{k_1 - k_2} S_B^{(2)} \tag{8}$$

$$D_1 = \frac{\beta k_1}{k_1 - k_3} S_C^{(2)} \tag{9}$$

Inversion of these linear equations yields the SADS, and ultimately the SAS are generated by adding a fraction of the CarH-GS spectrum (c_0). This therefore requires knowledge about c_0 and the branching fractions, α and γ (model 1) or α and β (model 2), used in each model. These parameters cannot be determined by a fit to the data. For each model, conversion to the SAS are therefore given as follows:

Model 0:

$$S_A^{(0)} = D_1 + D_2 + D_3 + c_0 S_{GS} \tag{10}$$

$$S_B^{(0)} = \left(\frac{k_1 - k_2}{k_1} D_2 - \frac{k_1 - k_3}{k_1} D_3 \right) + c_0 S_{GS} \tag{11}$$

$$S_C^{(0)} = \frac{(k_1 - k_3)(k_2 - k_3)}{k_1 k_2} D_3 + c_0 S_{GS} \tag{12}$$

Model 1:

$$S_A^{(1)} = D_1 + D_2 + D_3 + c_0 S_{GS} \tag{13}$$

$$S_B^{(1)} = \left(\frac{k_1 - k_2}{\alpha k_1} D_2 + \frac{k_1 - k_3}{\alpha k_1} D_3 \right) + c_0 S_{GS} \tag{14}$$

$$S_C^{(1)} = \frac{(k_1 - k_3)(k_2 - k_3)}{\alpha\gamma k_1 k_2} D_3 + c_0 S_{GS} \tag{15}$$

Model 2:

$$S_A^{(2)} = D_1 + D_2 + D_3 + c_0 S_{GS} \tag{16}$$

$$S_{\mathrm{B}}^{(2)} = \frac{k_1 - k_2}{\alpha k_1} + c_0 S_{\mathrm{GS}} \tag{17}$$

$$S_{\mathrm{C}}^{(2)} = \frac{k_1 - k_3}{\beta k_1} + c_0 S_{\mathrm{GS}} \tag{18}$$

As can be seen in eqs. 10, 13, and 16, the SAS of the first intermediate is given by the sum of all three DADS plus an appropriate amount, c_0, of the ground state spectrum in all three models. Different values of c_0 were therefore screened to assess the effect on the resulting shape of the SAS of $|\mathrm{A}\rangle$. To achieve a reasonable SAS the following two criteria were used: *i)* the SAS must not consist of negative spectral features; *ii)* the SAS should not contain significant spectral contributions from the CarH-GS spectrum. Supplementary Figs. 19a, 20a, and 21a show the SAS of $|\mathrm{A}\rangle$ using c_0 values ranging from 0.035 to 0.382 in 0.018 steps. Values of $c_0 < 0.035$ resulted in negative features in the SAS and therefore 0.035 represents the lower limit. For $c_0 < 0.199$ the SAS still showed the features of the ground state bleach, whereas > 0.199 the SAS started to resemble the CarH-GS spectrum (Supplementary Fig. 19d). Therefore, we assumed $c_0 = 0.199$ when generating the SAS of $|\mathrm{A}\rangle$ in all three models. In model **0** the other SAS only depend on the choice of c_0. However, using $c_0 = 0.199$ (as well as a range of other different values) results in almost identical SAS for $|\mathrm{B}\rangle$ and $|\mathrm{C}\rangle$ (Supplementary Figs. 19b and c), both of which are very similar to the spectrum of CarH-GS (Supplementary Fig. 19d). Therefore, model **0** was considered an inappropriate description of the data.

In model **1** the branching ratios α and γ also need to be chosen using the same constrains set out above for c_0. The SAS of $|\mathrm{B}\rangle$ only depends on the branching ratio α (eq. 14). Supplementary Fig. 20b shows the SAS of $|\mathrm{B}\rangle$ for different values of α, tuned from 0.0320 to 0.1916 in 0.0084 steps. $\alpha < 0.0320$ resulted in negative features of the SAS. $\alpha < 0.1076$ still shows highly unlikely spectral kinks at ~ 400, 425, 445, 525, 555, and 590 nm, whereas $\alpha > 0.1076$ the SAS started to resemble the CarH-GS spectrum (Supplementary Fig. 20d). Although the SAS of $|\mathrm{B}\rangle$ at $\alpha = 0.1076$ represents perhaps the optimum spectrum for this model, it still shows a high similarity to the CarH-GS spectrum. The SAS of $|\mathrm{C}\rangle$ in model **1** depends on both branching ratio α and γ (eq. 15), where α is already determined by generating the SAS of $|\mathrm{B}\rangle$. Fixing $\alpha = 0.1076$ and tuning γ from 0.283 to 0.774 in 0.026 steps gives SAS without negative features for $|\mathrm{C}\rangle$ (Supplementary Fig. 20c). $\gamma < 0.283$ the SAS still shows a highly unlikely spectral kink at ~ 590 nm. Above this value the SAS starts to resemble the spectrum of CarH-GS (Supplementary Fig. 20d). Therefore, $\gamma = 0.5157$ results in the most likely SAS of $|\mathrm{C}\rangle$, which closely resembles the pure spectrum of the MLCT state observed previously following the photoexcitation of methylcobalamin.[4] However, the similarity of

the SAS for |B⟩ (Supplementary Fig. 20b) to the CarH-GS spectrum (Supplementary Fig. 20d) means model **1** is also unlikely to accurately describe the data.

Model **1** was therefore modified to model **2**, allowing the additional branching from |A⟩ to |C⟩ and omitting the conversion from |B⟩ to |C⟩. In this model the branching ratios β and γ need to be chosen for |B⟩ and |C⟩, respectively. Supplementary Fig. 21b shows the SAS of |B⟩ for different values of β, tuned from 0.0128 to 0.0462 in 0.0018 steps. For values of $\beta < 0.0128$ the SAS shows negative features and for values of $\beta > 0.022$ the SAS starts resemble the CarH-GS spectrum (Supplementary Fig. 21d). At a value of $\beta \sim 0.022$, the SAS strikingly resembles the spectrum of the distinctive Co–C bond homolysis product, cob(II)alamin.[5] The SAS of |C⟩ was determined by using different values for γ tuned from 0.032 to 0.1441 in 0.00118 steps (Supplementary Fig. 21c). For values of $\gamma < 0.032$ the SAS has negative features and for $\gamma < 0.084$ the SAS shows a highly unlikely spectral kink at ~ 590 nm. However, at $\gamma \sim 0.084$ the SAS of |C⟩ closely resembles the pure spectrum of the MLCT state as of methylcobalamin.[4] One can therefore conclude that model **2** is the only model that is able to generate reasonable SAS which resemble known B_{12} spectra (*i.e.*, cob(II)alamin and the cob(III)alamin MLCT state).

Supplementary References

1 Díez, A. *et al.* Analytical ultracentrifugation studies of oligomerization and DNA-binding of TtCarH, a *Thermus thermophilus* coenzyme B_{12}-based photosensory regulator. *Eur. Biophys. J.* **42**, 463-476 (2013).

2 Drennan, C. L., Huang, S., Drummond, J. T., Matthews, R. G. & Ludwig, M. L. How a protein binds B_{12}: A 3.0 Å X-ray structure of B_{12}-binding domains of methionine synthase. *Science* **266**, 1669-1674 (1994).

3 Shiang, J. J. *et al.* Ultrafast excited-state dynamics in vitamin B_{12} and related cob(III)alamins. *J. Am. Chem. Soc.* **128**, 801-808 (2006).

4 Walker, L. A. *et al.* Time-resolved spectroscopic studies of B_{12} coenzymes: the identification of a metastable cob(III)alamin photoproduct in the photolysis of methylcobalamin. *J. Am. Chem. Soc.* **120**, 3597-3603 (1998).

5 Yoder, L. M., Cole, A. G., Walker, L. A. & Sension, R. J. Time-resolved spectroscopic studies of B_{12} coenzymes: influence of solvent on the photolysis of adenosylcobalamin. *J. Phys. Chem. B* **105**, 12180-12188 (2001).
